# Cercarial Dermatitis in Norway - An Emerging Zoonotic Disease

**DOI:** 10.1007/s11686-025-01083-2

**Published:** 2025-07-04

**Authors:** Arnulf Soleng, Thora Gundersen, Heidi Lindstedt

**Affiliations:** 1 Department of Pest Control, Norwegian Institute of Public Health, Oslo, Norway; 2 Present Address: Department of Virology , Norwegian Institute of Public Health, Oslo, Norway; 3 Present Address: The University of Oslo, Oslo, Norway

**Keywords:** Avian schistosomes, Cercariae, Flukes, Geographical distribution, Schistosomatidae, Swimmer’s itch

## Abstract

**Objective:**

Cercarial dermatitis, often called swimmer’s itch, is a non-communicable skin condition caused by bird schistosomes. The host response is characterized by an early type I hypersensitivity reaction and a late phase of cutaneous inflammation, often leading to severe itching. Outbreaks of cercarial dermatitis in recreational waters can significantly impact public health. The aim of this present study was to describe the geographical distribution of reported cercarial dermatitis in Norway from 1980 to the end of 2023.

**Methods:**

The study is based on random reports obtained from private persons, hospitals, general practitioners and environmental health sectors experiencing the problem.

**Results:**

The first case of cercarial dermatitis in Norway was reported back in 1980. In the following years, an increasing number of cases were reported with a clear increase from 2010 and onwards. By the end of 2023 cercarial dermatitis was reported from a total of 414 lakes and 37 rivers. Approximately 81% are from freshwater below 300 m elevation, and 90.9% are from areas located south of the Arctic Circle at 66.3°N. As far as we know, the case report from 70.4°N is the northernmost in Europe.

**Conclusions:**

The observed increased geographical distribution of cercarial dermatitis in Norway might be explained by increased water temperatures, more infected migrating birds, more suitable environmental factors facilitating snail and fluke survival, as well as increased attention in media, internet and social networks. Identification of the causative agent or agents and their geographical distribution is important for estimating the potential health risk and for designing appropriate preventive and control measures for this emerging disease in Norway.

**Supplementary Information:**

The online version contains supplementary material available at 10.1007/s11686-025-01083-2.

## Introduction

The association between avian schistosomes and cercarial dermatitis in humans has been known for many decades. Cercarial dermatitis (swimmer’s itch) is a non-communicable skin condition caused by bird schistosomes in the trematode family Schistosomatidae. The host response is characterized by an early type I hypersensitivity reaction and a late phase of cutaneous inflammation, both associated with a polarized Th2-type acquired immune response [1]. A recent study of host response to *Schistosoma mansoni* revealed a previously unrecognized intercellular communication mechanism wherein itch-inducing MrgprA3 neurons initiate host immunity against such skin-invasive cercarial parasites [2]. Haas and Van de Roemer [3] reported that the similarity of skin lipid composition between aquatic birds and humans may be a factor that contribute to the erroneous penetration of human skin by bird schistosomes. The clinical symptoms of cercarial dermatitis were first described in 1928 [4], and include intensive itching, maculae, papulae, urticariae and, in some cases, limb swelling, local oedema with enlarged lymphatic nodes, fever, nausea and diarrhoea [5, 6]. Cercarial dermatitis develops more rapidly and presents with more severe symptoms in individuals with a prior history of the condition, although symptom onset also occurs, to a lesser extent, in naive persons with no previous exposure [7]. Thus, repeated exposure often leads to more severe symptoms with a more pronounced cutaneous reaction followed by diffuse edema and development of erythematous papules or papulovesicles [8, 9].

Cercarial dermatitis is reported from every continent except Antarctica [10]. Reports are common from lakes in North America [11–13]. In Europe, cercarial dermatitis is especially frequent in lakes in the temperate regions [14], including UK [15]. But also, northern Europe is affected by this zoonosis, e.g., Denmark [16–20], Sweden [21], Finland [22, 23], Iceland [24, 25] and Norway [26].

Cercarial dermatitis is considered an emerging disease in several European countries [25, 27, 28], and its public health impact needs to be evaluated globally [27]. The Norwegian Institute of Public Health (NIPH) have performed yearly surveillance of the occurrence of cercarial dermatitis in Norway since the first verified report back in 1980. This surveillance is mainly based on reports to the institute from persons suspecting cercarial dermatitis. Data concerning the geographical distribution of cercarial dermatitis during the 30-year period from 1980 to 2009 in Norway is previously published [26] and showed a steady increase from one lake in 1980 to 87 lakes, ponds and rivers by the end of 2009. Based on retrospective reports, some new lakes with cercarial dermatitis have been included in the data from the period 1980 to 2009 in the present study.

Outbreaks of cercarial dermatitis in recreational waters can significantly impact public health. Environmental monitoring and surveillance are important for outbreak prediction and public health management. Thus, the main aim of this present study was to describe the geographical distribution of reported cercarial dermatitis in Norway from 1980 to the end of 2023.

## Materials and Methods

In Norway, there is currently no mandatory reporting system for cercarial dermatitis, nor is there any legislation requiring the surveillance of cercarial dermatitis or avian schistosomes in recreational freshwater environments. The data in the present study is based upon random cases reported to NIPH during the 44-year period from 1980, when the first case was reported in Norway, until the end of 2023. Reports came from several sources, e.g., general practitioners, physicians, hospitals, environmental health officers in different municipalities, but the majority came from the public. During the first two decades most reports came by phone or in some cases by fax. After the millennium more and more reports came by e-mail, but also some by phone calls, especially from elderly people. From the year 2009 and onwards, people who experienced what they believed to be swimmer’s itch were encouraged on the NIPH website (https://www.fhi.no/sm/badevann/svommekloe/) to report this to the institute.

Most case reports to NIPH included several affected persons (typically families with children). They often reported an itchy rash after swimming and bathing, and they were suspecting cercarial dermatitis, but in some cases, they were unaware of this disease. The diagnosis cercarial dermatitis was assessed by the anamnesis as the persons reporting the dermatitis was interviewed by phone or e-mail to eliminate other possible causes of the skin rash, such as bites from insects and ticks, stinging nettles, algae, bacteria etc. The interviews were conducted by two persons; Reidar Mehl in the period 1980 to 1999, while Arnulf Soleng conducted the interviews from 2000 to 2023. Cercarial dermatitis was suspected when the affected persons reported a tingling, itching sensation of the skin immediately after swimming, and/or subsequently experienced small red itching parts of the skin that reached its maximum after about 24 h or later. In many cases the report also included photographs of the skin rash making the correct diagnosis easier. Furthermore, when the correct clinical symptoms only appeared on parts of the body which had been in direct contact with water, was evenly distributed over the exposed skin, and did not appear on parts covered by watertight swimsuits like neoprene suits, cercarial dermatitis was diagnosed. The name and the location (UTM-coordinates, county, and municipality) of the lake, pond or river where the bathing took place was noted. No serological studies were performed, and no cases were diagnosed by a dermatologist during this study. In the period 2020 to 2023 we asked for the age of the affected persons, although this was not a main aim of the surveillance.

We had the following exclusion criteria of the case reports: Data were omitted from the study when the interview made it doubtful that cercariae was the cause of the skin rash. Furthermore, in cases where it was impossible to perform the interview (e.g., anonymous report or no response to phone calls or e-mails), and when the persons could not state the name of the exact lake where the exposure took place, the data was excluded.

## Results

**Fig. 1:**
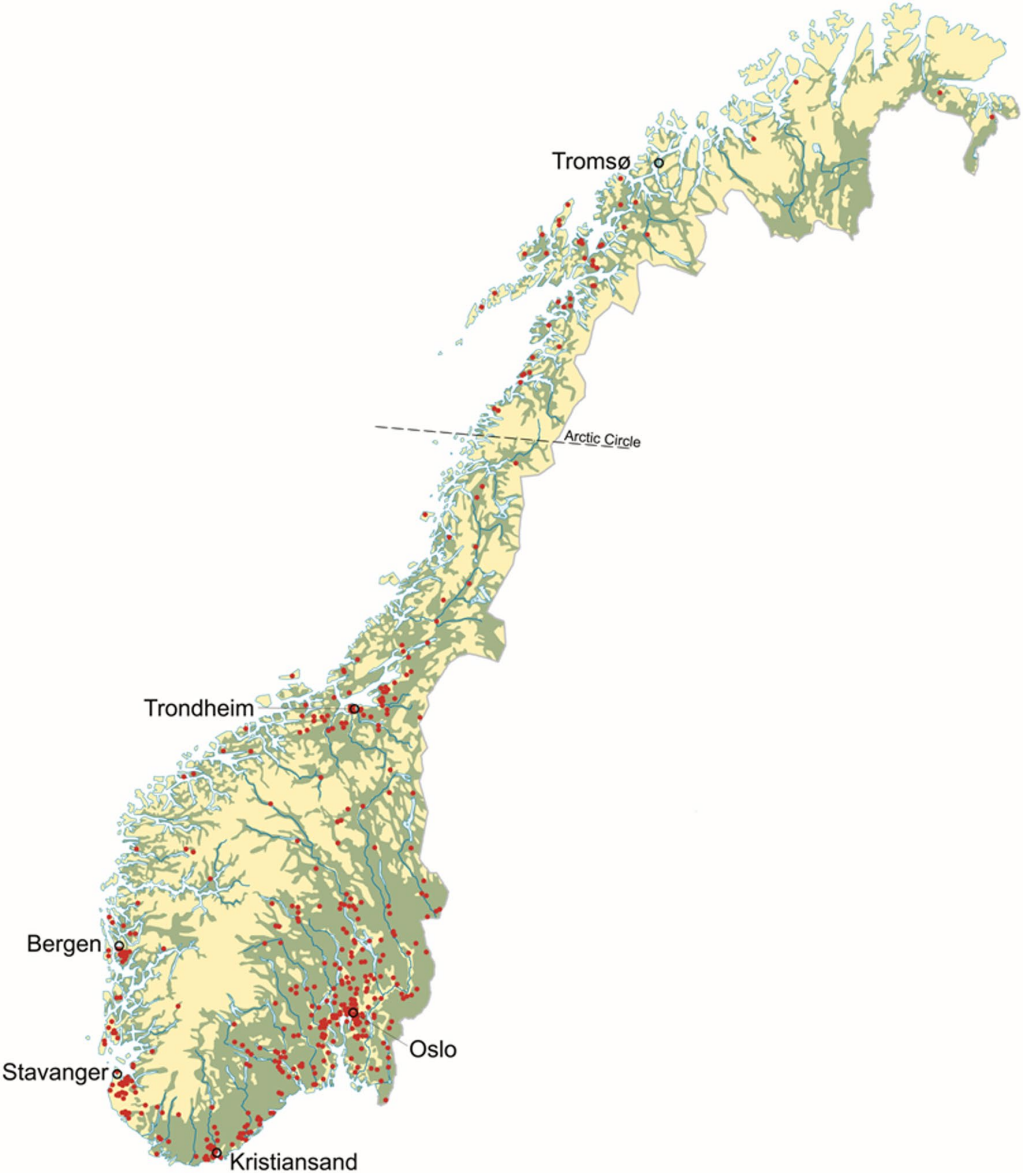
Geographical distribution of reported cercarial dermatitis in Norway, 1980–2023. Note that each red dot might represent several lakes, ponds, and rivers

**Fig. 2:**
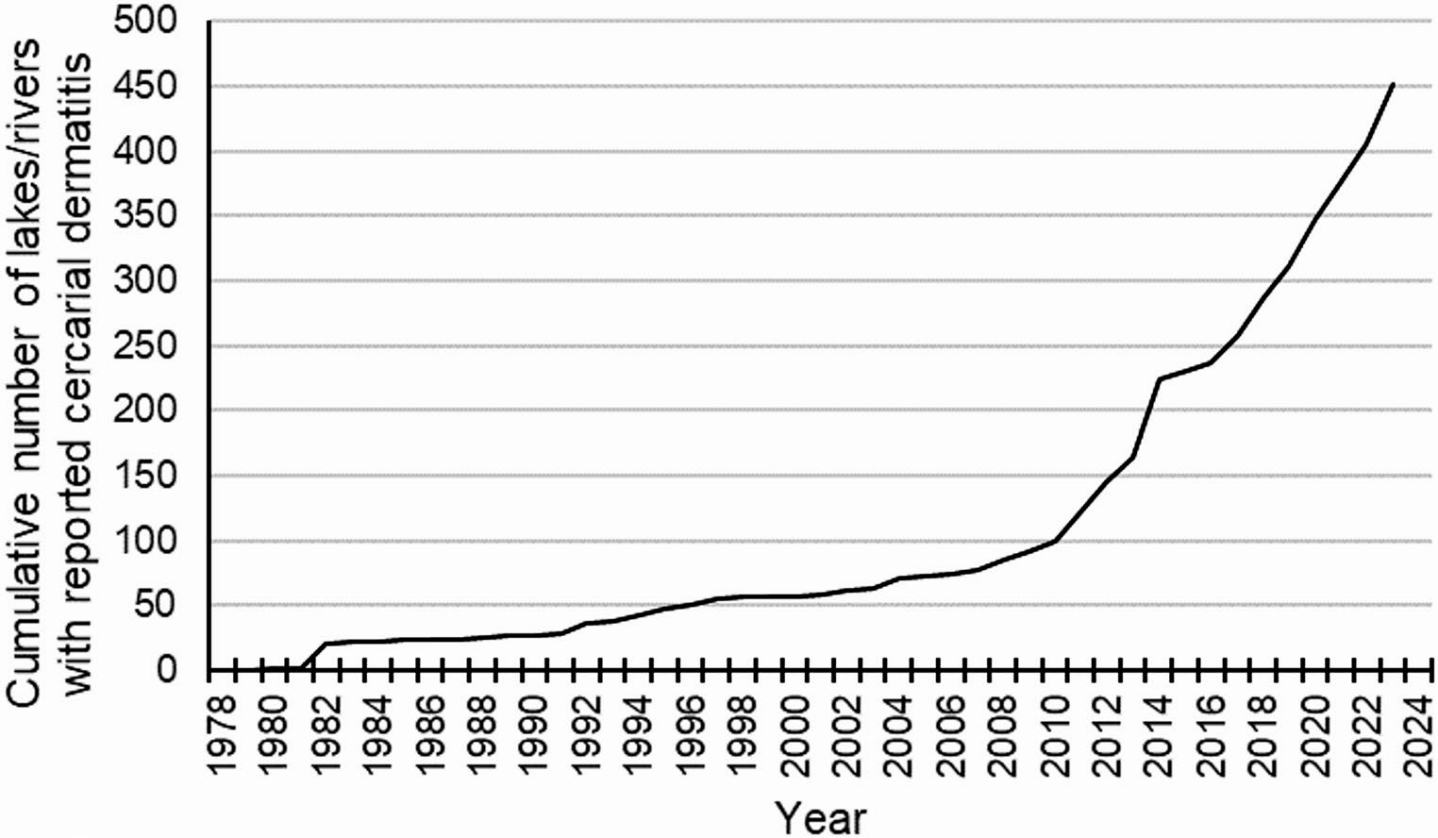
Cumulative number of lakes and rivers with reported cercarial dermatitis in Norway during the 44-year period since the first report in 1980 to the end of 2023. Data from 1980 to 2009 is previously published by Soleng and Mehl [5], but in retrospect a few more lakes are included

**Fig. 3:**
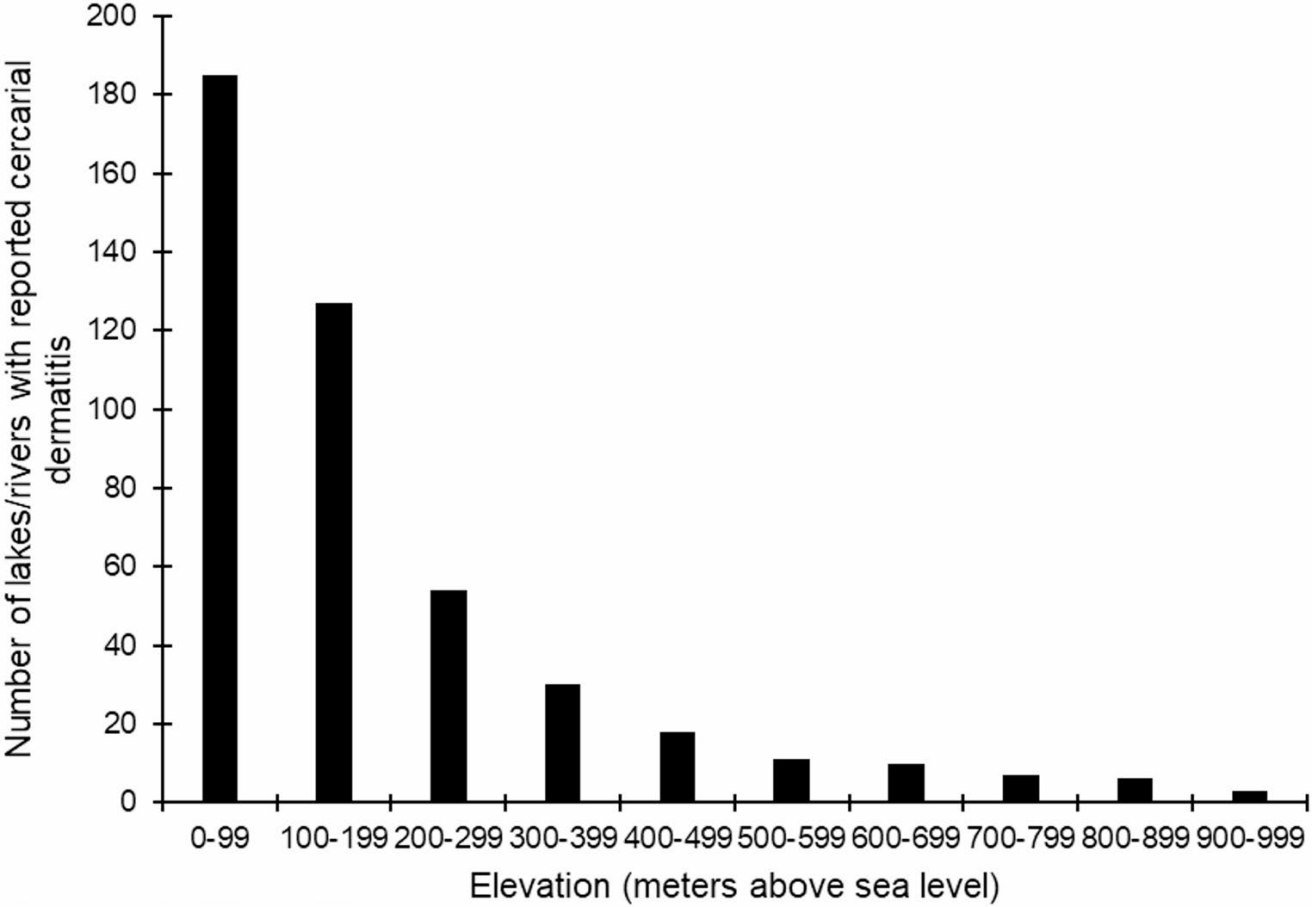
Number of lakes and rivers with reported cercarial dermatitis in Norway compared to elevation (meters above sea level). Note that approximately 81% of reports came from lakes and rivers below 300 m above sea level

**Fig. 4:**
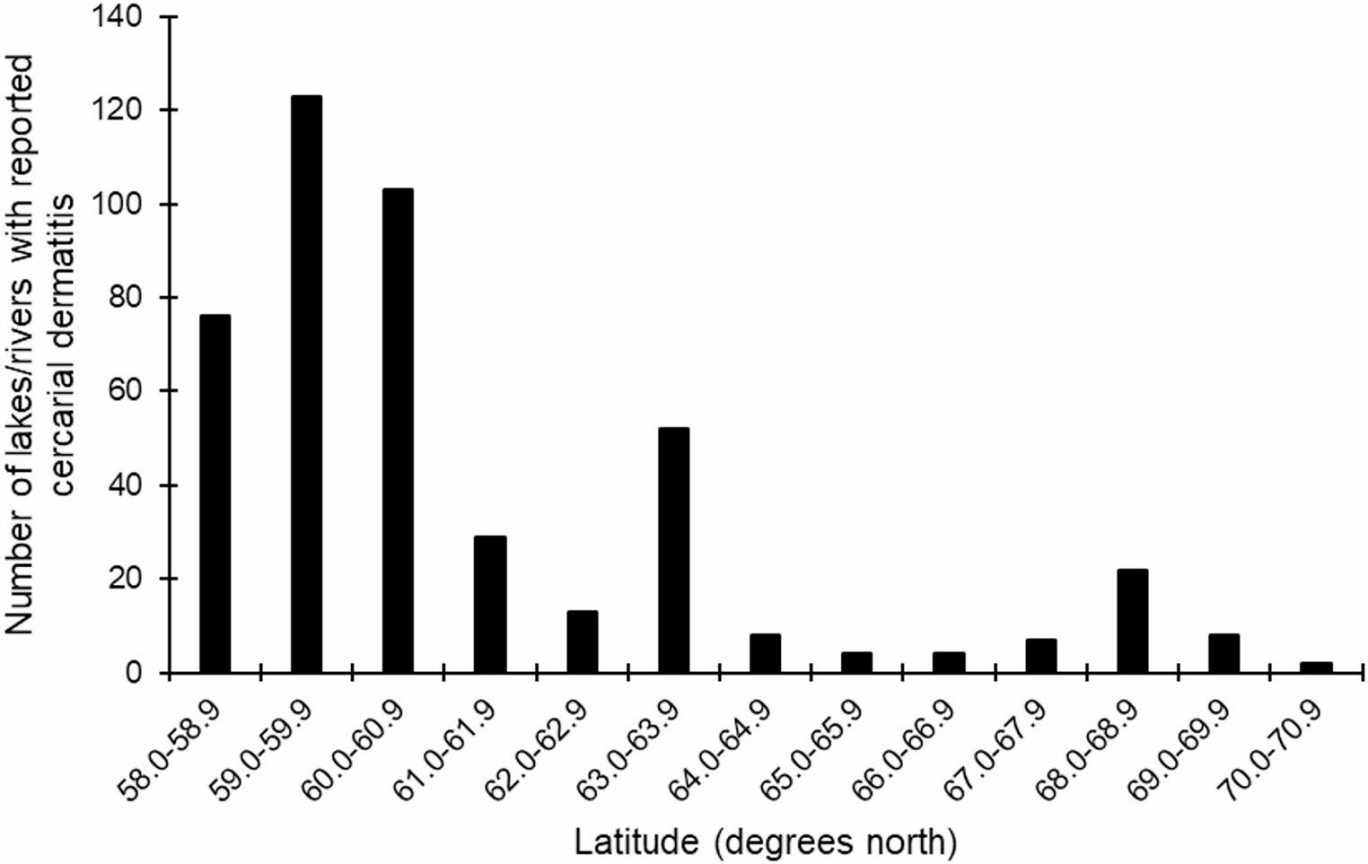
Number of lakes and rivers with reported cercarial dermatitis in Norway compared to latitude (degrees north). Note that the Arctic Circle is located at 66.3°N

By the end of 2023 cercarial dermatitis was reported from 414 lakes and 37 rivers, a total of 451 freshwater localities (cf. supplementary Tables 1 and Figs. 1 and 2). These freshwater habitats were in 211 of the 357 municipalities in Norway. Rivers might run through several communities and more than one county. Thus, in many rivers we have reports from several locations in several communities and in some cases from more than one county. However, the location of the first report is presented in supplementary Table 1. Furthermore, this zoonosis is reported from all 15 counties in Norway, and reports came from lakes with different ecological and chemical status (cf. supplementary Table 1). Lakes were located close to the sea level and even as high as 937 m above sea level (Fig. 3). Approximately 81% of the reports are from areas below 300 m elevation. Almost 67% of the reports are from the southern latitudes between 58.0 and 60.9°N, and 90.9% from areas located south of the Arctic Circle at 66.3°N. The northernmost report came from a lake located at 70.4°N in Finnmark County (Figs. 1 and 4).

Areas with most reports are from population rich areas in the southern part of the country at lower elevations (Figs. 1, 3 and 4) where the climate is warmer. In the period from 2010 to 2023 there is a clear increase in number of freshwater locations with reported cercarial dermatitis, with changes both with elevation and latitude compared with the earlier period from 1980 to 2009. Some lakes have yielded occasional reports, while some have had just a single report in a single year. In 68 of the 451 lakes/rivers where the disease is reported, we have received reports almost every single year after the first occurrence of cercarial dermatitis. The latter lakes are marked with an asterisk (*) in the supplementary Table 1. Note that we have identified ocellate cercaria emerging from snails collected in 12 different lakes. These lakes are marked with ^π^ in the supplementary Table 1.

In the period from 1980 to 2019 we did not register the age of the affected patients. However, in the period from 2020 to 2023 we asked for the age when this was not stated in the first report. Some people were reluctant to answer this, and others just used the term “small children”, “children”, “teenagers” or “adults” instead of specifying the exact age. Nevertheless, all age groups were found to be affected, ranging from around 1 year to over 80. Between 2020 and 2023, we received 1086 case reports involving a total of 1559 individual patients. Children younger than 13 years accounted for 65.1% of all these patients, and 9.2% were teenagers. Thus, people under the age of 20 years accounted for 74.3% of all patients with cercarial dermatitis. Adult people above 20 years accounted for 25.7% of persons with cercarial dermatitis.

Furthermore, during the latter years we received some reports of swimmer’s itch in some specific lakes in southern Norway as long back as the mid-1970s, but with no photographical evidence and without recognition of the etiology of the condition. We have not included such unverified data in this study. Approximately 8% of the reports we received in the whole period where excluded based on the exclusion criteria. We have also some reports of cercarial dermatitis from brackish water (8 cases) and sea water (6 cases) in the southern part of Norway. These cases are, however, not verified as swimmer’s itch.

## Discussion

Cercarial dermatitis is considered an emerging zoonosis in Europe [25, 27, 28]. Defining an emerging disease is not straightforward, as there are several different types of disease emergence [29]. The emergence of bovine spongiform encephalopathy in the 1980s is an example of a brand-new disease. A geographic emergence in an area not previously affected, such as the emergence of bluetongue in northern Europe in 2006 is another example. There can also be an unexpected increase of disease incidence in a known area and a known species, or there may simply be an increase in our knowledge or awareness of a particular disease. Nevertheless, cercarial dermatitis might be an emerging disease in Norway based on the present data. However, several factors can explain why we have recognised a strong increase in the number of case reports of cercarial dermatitis in Norway, especially from 2010. Changes in climate, better water quality for the intermediate snail hosts and free-swimming stages, migration of infected birds from southern areas in Europe etc. An increased focus on the problem in media automatically increases the number of phone calls and e-mails from people and physicians suspecting cercarial dermatitis as noted by Soleng and Mehl [26]. The Internet has made it easier for people to find information about cercarial dermatitis, and people might feel that the threshold for contacting NIPH is lower when using e-mail in recent years, as compared to phone calls (and fax) which were more common before the millennium. Furthermore, over the last 15 years information on the institute’s website has encouraged members of the public and physicians to report cases of cercarial dermatitis. Frequent coverage in newspapers, on radio and TV and on different social media platforms might also have contributed an increase in reports.

The first case report of cercarial dermatitis in Norway came back in 1980 from Lake Jonsvatnet near Trondheim in the middle of Norway [26]. Soleng and Mehl [26] described the occurrence and geographic distribution of the disease in Norway from this first report until 2009 when cercarial dermatitis was reported from 89 lakes. In retrospect, cercarial dermatitis was reported from 5 more lakes, thus totalling 94 by the end of 2009. In the period from 2010, and until the end of 2023 there is a clear increase in number of locations with cercarial dermatitis, with changes both with elevation and latitude compared with earlier results. As the present data is based on random reports, the geographical distribution presented in this study can be misleading as the disease could have been misidentified despite thorough interviews. Differential diagnosis of cercarial dermatitis is difficult since several different agents can cause dermatitis [11], e.g., bites from ticks, bed bugs, mosquitoes and black flies, stings from stinging nettles, exposure to different algae and bacteria. However, it is also likely that many cases of swimmer’s itch go unreported as people are unaware of our surveillance. Therefore, we believe that the geographical distribution is in fact underestimated. Based on the case reports, cercarial dermatitis is distributed nearly all over Norway, from sea level and up to above 900 m above sea level, and from the south to the north. The case report in Finnmark County at 70.4°N are, to our knowledge, the northernmost report of cercarial dermatitis in Europe. Approximately 81% of the reports came from areas below 300 m elevation, and 67% of the reports are from the southern latitudes between 58.0 and 60.9°N. This latter area consists of 35% of the total Norwegian land mass and 42% of freshwater lakes within the country. It is also evident, and not surprising, that most reports are from population rich areas around cities like Oslo, Drammen, Kristiansand, Stavanger, Bergen and Trondheim. In conclusion, most reports originate from southern Norway, particularly in densely populated areas at lower elevations and around lakes commonly used for recreational activities.

The mainland part of Norway spans latitudes from approximately 58°N to 71°N. The country shows a complex combination of physical factors leading to various climate settings. Due to the huge extension in north–south direction Norway encompasses five climate zones according to the Köppen classification [30, 31]. Due to its location close to the North Atlantic Current the climate is more temperate compared to what is to expect from the given geographical zone [32]. The southwestern coastal lowlands experience a relatively temperate climate. In contrast, the mountainous regions just inland receive substantial precipitation throughout the year, often including heavy snowfall in winter that sustains numerous glaciers. Meanwhile, the more continental areas of Eastern Norway tend to be much drier. The elevated mountain plateaus, particularly in northern Norway, exhibit subarctic characteristics, including the presence of permafrost [32]. However, climate change is affecting Norway. From the normal period 1961–1990 to the current normal period 1991–2020, the annual average temperature in Norway has increased with approximately 1 °C [33, 34]. The transition seasons have become longer in many coastal areas and shorter in some inland areas. On average there have been more Nordic summer days (days per year with max temperature ≥ 20 °C), heat waves with maximum temperature ≥ 27 °C in five consecutives 24-hour periods and tropical nights (nights between 8PM and 8AM with temperatures ≥ 20 °C) [35], and the geographical areas where these occur has become larger [33]. The length of the growing season has also increased [33]. Especially since 1980, a marked temperature increase is observed over the whole country [33, 34]. Lake surface temperatures have increased in the last decades on a global scale in line with increasing air temperatures [36]. Surface water warming rates are dependent on combinations of climate and local characteristics, rather than just lake location [37]. The widespread warming reported by O´Reilly et al. [37] suggests that large changes in Earth’s freshwater resources and their processes are not only imminent but already under way. Global warming can affect the world’s biota and the functioning of ecosystems in many indirect ways, and climate change might alter the geographical distribution of parasitic diseases [38]. Thus, climate changes might have increased the problem with cercarial dermatitis in Norway due to the warming of lake surface waters. Global warming is considered an important risk factor for parasite prevalence in lakes, as elevated temperatures can provide more favorable conditions for birds, schistosomes and snail hosts [39]. Furthermore, other environmental factors might also have contributed. In the 1980s and 1990s, acidification was a major environmental problem in Norway. Southern Norway was heavily influenced by freshwater acidification, leaving a lot of lakes deprived of fish and invertebrates [40–43]. Freshwater snails are intermediate hosts for avian schistosomes, and they are known to be even more sensitive than fish to low pH values [44]. Subsequently, many lakes in southern Norway have earlier been deprived of the snail intermediate hosts, making it impossible for the avian flukes to complete their life cycle. Fortunately, emissions of sulfur and nitrogen compounds in Europe have been significantly reduced over the past 30 years being lowered by over 90% since 1990 [45]. Currently, 6% of Norway’s land area receives more sulfur and nitrogen compounds than the environment can handle. By comparison, in 1990 this figure was 26%. The areas where the critical load is exceeded are primarily in southern and southwestern Norway [45]. Water snails and free-swimming organisms in general are influenced by abiotic environmental factors. For example, a study on monogeneans demonstrated that a particular ectoparasite on Atlantic salmon was very sensitive to aqueous aluminium and low pH caused by acidic rain [46]. The free-swimming miracidium and cercariae of blood flukes might also be more sensitive than freshwater snails to these environmental factors, thus limiting the survival and existence of these free-swimming stages even in places where snails are capable of living. Government-funded liming (adding lime to neutralize acidity) is an important environmental measure in southern Norway. The effort began in the 1980s and expanded throughout the 1990s and into the early 2000s. Today, 24 salmon rivers and approximately 1,000 lakes are limed [45]. Soleng and Mehl [26] suggested that as acidification problems decline, there could be an increase in both the number and distribution of potential snail hosts, improved survival of free-swimming miracidia and cercariae, and a general rise in bird populations as freshwater ecosystems recover. However, there is no comprehensive, long-term systematic monitoring of freshwater snails in Norway that can track population changes from 1980 to the present. Nevertheless, the environmental conditions are becoming more suitable for the snail intermediate hosts for avian schistosomes, possibly making the problem of cercarial dermatitis worse. It is noted that increased contact of people with schistosome-infested water, high eutrophication of water reservoirs, colonization of ponds by susceptible snails and nesting ducks and long periods of sunshine in the summer are important factors that have led to recent increases in the number of outbreaks of cercarial dermatitis in some areas in Europe [47].

Horák et al. [5] suggested that cercarial dermatitis is most prominently distributed along the major flyways of migratory birds. In many of the case reports from Norway people mention that different species of waterfowl (Anseriformes) and to some extent gulls (Charadriiformes) were present at the lakes at the time of the swimming. However, it is not necessary for the birds to be permanently present to establish the parasites in the intermediate snail hosts in the lake, as parasite eggs can be released during the transient presence of infected migrating birds. The prepatent period in snails is about 3 to 10 weeks [5] and can be longer if the water is cold as it often is in northern Norway and at higher elevations. For the waterfowl schistosome *Trichobilharzia* species, it seems that the spectrum of final bird hosts is broader than that of the intermediate snail hosts, and it is also clear that these fluke species can infect bird species other than waterfowls [5].

The total number of case reports from each lake was variable as also Soleng and Mehl [26] reported. Of the 451 lakes and rivers with reported cercarial dermatitis, 68 have provided frequent reports almost every year since the initial case report. Other lakes have yielded only occasional reports, while some have had just a single report in a single year. Thus, the disease seems to be endemic in some lakes, whereas in others it seems to appear more sporadically. Schistosomes can survive in overwintering snails, serving as a source of infection in the spring [48], but if this happens in snails in Norwegian lakes is not known. Eutrophication can increase the growth of snail populations and transmission of bird schistosomes [48]. In a study of the trophic status of 366 lakes in Norway, it was found that most of the eutrophic lakes are in the water regions of Eastern Norway, as well as in the water regions of the counties Rogaland and Vestland on the southwest coast [49]. These areas are often influenced by agricultural runoff and scattered sewage, as well as some leakage from sewage networks and overflow from municipal wastewater treatment plants. Oligotrophic lakes are prevalent at higher altitudes in southern Norway and are also commonly found in the northern counties of Nordland, Troms, and Finnmark [49]. Although we don’t have the trophic status of all lakes affected by cercarial dermatitis in Norway, we can conclude that cercarial dermatitis occur in lakes of different environmental status. A total of 75% of the lakes where the disease appears to be endemic is situated south of 62°N, and 90% of these are situated below 300 m above sea level– in areas with more eutrophication and warmer climate. However, these endemic lakes exhibit variability in both ecological and chemical status, with no discernible pattern or consistent trend regarding the presence of cercarial dermatitis (cf. supplementary Table 1). However, all these lakes are very popular recreational areas, typically located in densely populated areas. The differences in case reports between lakes can also be explained by the variation in water temperature in some cases, especially the sporadic reports from lakes at higher elevations and in North Norway. These lakes are typically very cold, which may prevent the flukes from completing their life cycle in most years. Environmental temperature is an important factor influencing both the development and transmission of these avian flukes. Furthermore, at low temperatures the exposure of humans to cercariae is also very limited, as there is a positive correlation between frequency and duration of bathing and increasing water temperature. In the study presented here, reports of swimmer’s itch appeared very frequently when the water temperature reached 20 °C and above in in the months of June, July, and August. Thus, water temperature is an important factor explaining the incidence of swimmer’s itch. In the warm summer months, the increased release of cercariae from the intermediate snail hosts coincides with people’s increased contact with freshwater during bathing, swimming, and recreational water sports. This seasonality is in accordance with other studies [5, 22]. A multivariate analysis [9] showed that the time of day, barometric pressure, maximum air temperature and duration of swimming activity influence not only the incidence of cercarial dermatitis, but also the number of skin lesions. The early study of Neuhaus [50] showed that free-swimming schistosome cercariae were released from snails in large numbers especially on sunny days. In a study from Denmark, Tracz et al. [51] state that shedding of cercariae from snails is temperature dependent, and high temperatures and sunshine increase the risk of encountering the parasite and acquiring cercarial dermatitis.

In addition to variations in case reports between different lakes, we also observed differences between specific areas within the same lake when such data were available. This might be due to other general environmental conditions. Snails might be more abundant in certain parts of the lakes, especially in areas with much aquatic vegetation. Wind exposure and water currents also cause cercariae to accumulate in certain areas of the lakes, leading to great differences in human exposure to the parasite depending on where people bath, swim or do their water sports. In a study in from Michigan swimmers faced the highest risk of cercarial dermatitis when entering the water in the morning or on days with direct onshore winds blowing perpendicular to the shoreline [52]. However, after adjusting for wind direction, increased wind speed had a negative association with infection risk, with the highest risk occurring on days with a gentle offshore breeze. These findings suggest that at this beach in Michigan, direct onshore winds create surface-water currents that concentrate cercariae in the shallow areas frequented by swimmers [52]. Furthermore, Leighton et al. [12] used plastic markers to measure surface water currents and suggested that large concentrations of cercariae could accumulate at the surface in hyperendemic areas and subsequently be transported into swimming areas of the lake. Such concentrations of cercariae, leading to hyperendemic zones, may help explain why reports of cercarial dermatitis vary from day to day at the same lake and differ significantly across various locations within the lake.

It is recognised that cercarial dermatitis affect all age groups [9]. However, children between 5 and 9 years of age are at the highest risk because their exposure to water during recreational activities is highest [53]. This was also evident in the present study, where individuals under the age of 20 accounted for 74.3% of all diagnosed patients of cercarial dermatitis in the period from 2020 to 2023. Children under 13 accounted for 65.1% of all patients. It is known that young children often spend a lot of time in shallow areas of beaches, where cercariae may gather in high concentrations, whereas teenagers and adults usually swim and play in the deeper parts of the lakes. Schets et al. [54] also reported that longer and more frequent exposure to freshwater resulted in an increase in reported symptoms of swimmer’s itch among children. Children are also less likely to dry their skin with towels, possibly increasing the chances of cercarial penetration. Parents might also be more eager to report swimmer’s itch when children are involved. Nevertheless, in the present study all age groups were found to be affected, ranging from around one year to over 80. Adult persons who reported cercarial dermatitis, often reported the typical symptoms with a tingling, itching sensation of the skin immediately after swimming. This was not so notably among the younger children. Some children were too young to express such skin symptoms, and some might have been occupied with playing in the water and thus not noticed the symptoms before the itching got severe. Many parents could often state retrospectively that they had noticed some discomfort among the children before the maculopapular lesions appeared.

In retrospect, reported cases of itching skin rash after freshwater bathing have appeared several years before 1980 in a few lakes without recognition of the etiology of the condition. In Iceland, retrospective reports indicate that swimmer’s itch has affected humans for almost a century [25], thus the problem might have been present in Norway years before 1980.

We have also some reports of cercarial dermatitis from brackish water (8 cases) and sea water (6 cases), but these cases are at present not diagnosed as cercarial dermatitis. Nevertheless, cercarial dermatitis is reported from brackish and sea water in other parts of the world [5, 55, 56], and studies should be performed in Norway to clarify this issue.

Confirming the presence of the zoonotic disease agents following reports of human infections in recreational waters should be demonstrated to verify that avian schistosomes are involved. Information about the most important snail and bird hosts and their fluke species is virtually absent in Norway, although unidentified schistosomatid ocellate cercariae have been found in snails sampled from 12 lakes (cf. Supplementary Table 1). So far, only one fluke species (*Trichobilharzia franki*) from a single infected snail (*Radix auricularia*) has been identified in Norway [26]. The possibility of using eDNA as a simple method to identify cercariae [57, 58] should be considered as identification of the causative agent is important for estimating the potential health risk and designing appropriate preventive and control measures [5].

## Conclusions

Although cercarial dermatitis is most frequently reported from lowland areas in the southern parts of Norway, the disease is also reported from higher elevations and from northern Norway. Since the first reported case in 1980, we now document a rise in the number of lakes, rivers, and ponds affected by cercarial dermatitis, totalling 451 freshwater locations by the end of 2023. The increase in new lakes is especially notable from 2010 and onwards. Thus, this indicate that cercarial dermatitis is an emerging disease in Norway. The increased geographical distribution of these avian flukes causing swimmer’s itch might be explained by increased water temperatures, more infected migrating birds, better environmental factors in the freshwater systems facilitating snail and fluke survival. Furthermore, increased attention of the problem in media, internet and social networks is probably an important factor which have contributed to more case reports. Identifying the causative agent(s), key snail and bird hosts, along with conducting detailed surveillance of the occurrence and geographical distribution of these avian schistosomes, is crucial from a public health perspective for assessing potential health risks and developing effective prevention and control strategies.

## Electronic Supplementary Material

Below is the link to the electronic supplementary material.

Supplementary Material 1

## Data Availability

No datasets were generated or analysed during the current study.
