# Supplementary material for: Cercarial Dermatitis in Norway - An Emerging Zoonotic Disease
Source: Acta Parasitol. 2025 Jul 4;70(4):143. doi: 10.1007/s11686-025-01083-2 (PMC12227355; doi:10.1007/s11686-025-01083-2)
Supplement: Supplementary file 1 — Supplementary Material 1 [file 11686_2025_1083_MOESM1_ESM.docx]

**Supplementary Table 1** Lakes and rivers with reported cercarial dermatitis in the period from 1980 to 2023. Year is the time for the first report to the Norwegian Institute of Public Health. Thus, there can be reports from the same lake in many subsequent years. Some lakes and rivers cross county and municipal boundaries, and they will therefore appear more than once in the table. The UTM coordinates refers to the lake/river and not the exact location where the swimming or bathing took place. Ecological and chemical status refers to environmental status of the lake; VG= very good, G=good, M=moderate, B=bad, VB=very bad, - = not classified (cf. https://vann-nett.no/waterbodies/map).

| **County** | **Lake/river** | **Municipality** | **UTM 33 (EUREF89)** | **Elevation**  **(MASL)** | **Latitude** | **Year** | **Ecological / chemical status** |
| --- | --- | --- | --- | --- | --- | --- | --- |
| Agder | Barbulia | Risør | 166313E 6523740N | 59 | 58.4°N | 2022 | (-/-) |
| Agder | Birketveitstjønna* | Iveland | 86760E 6501439N | 200 | 58.5°N | 1997 | (-/-) |
| Agder | Bjellandsvannet | Arendal | 146168E 6505539N | 6 | 58.5°N | 2010 | (M/B) |
| Agder | Boråstjenn | Froland | 127530E6502812N | 61 | 58.3°N | 2022 | (-/-) |
| Agder | Bråvann | Kristiansand | 83899E 6463304N | 67 | 58.1°N | 2020 | (-/-) |
| Agder | Byglandsfjorden* | Bygland/Evje og Hornnes | 83252E 6529241N | 207 | 58.6°N | 2012 | (M/B) |
| Agder | Båssviktjenna | Risør | 158462E 6523444N | 23 | 58.7°N | 2014 | (-/-) |
| Agder | Donevannet | Søgne | 82129E 6459555N | 8 | 58.1°N | 2019 | (-/-) |
| Agder | Eiketjønna | Søgne | 75756E 6461578N | 24 | 58.1°N | 2010 | (-/-) |
| Agder | Farvatnet | Kristiansand | 80641E 6467308N | 26 | 58.1°N | 2011 | (-/-) |
| Agder | Fjæretjenn | Tvedestrand | 147505E 6514299N | 21 | 58.4°N | 2023 | (-/-) |
| Agder | Førlandsvatn | Kvinesdal | 32736E 6486197N | 248 | 58.2°N | 2023 | (-/-) |
| Agder | Gillsvannet | Kristiansand | 90227E 6470819N | 1 | 58.1°N | 2022 | (M/B) |
| Agder | Gjennestadtjenna | Arendal | 130402E 6494525N | 60 | 58.4°N | 2014 | (-/-) |
| Agder | Grimevann | Lillesand | 112300E 6481488N | 43 | 58.2°N | 2020 | (M/B) |
| Agder | Grimsvann | Kristiansand | 83856E 6464404N | 54 | 58.1°N | 2017 | (-/-) |
| Agder | Grunnetjønn | Vennesla | 81687E 6478158N | 250 | 58.2°N | 2013 | (-/-) |
| Agder | Hagelandsvann | Vennesla | 73944E 6487359N | 311 | 58.2°N | 2021 | (-/-) |
| Agder | Hanangervatnet | Farsund | 13006E 6467677N | 2 | 58.1°N | 2013 | (M/-) |
| Agder | Hauslandstjenna | Grimstad | 129571E 6488354N | 54 | 58.2°N | 2022 | (-/-) |
| Agder | Hestvannet | Kristiansand | 83581E 6475185N | 105 | 58.2°N | 2014 | (-/-) |
| Agder | Hoksvannet | Kristiansand | 94465E 6463116N | 2 | 58.1°N | 2021 | (-/-) |
| Agder | Kilandsvannet | Grimstad | 114881E 6493118N | 161 | 58.4°N | 2019 | (G/-) |
| Agder | Lindvatnet | Sirdal | 16486E 6529958N | 376 | 58.6°N | 2012 | (B/-) |
| Agder | Lona | Vennesla | 81710E 6478834N | 206 | 58.2°N | 2011 | (B/-) |
| Agder | Lygnevannet | Hægebostad | 41917E 6518919N | 185 | 58.5°N | 1995 | (-/-) |
| Agder | Meldalstjenn | Tvedestrand | 143850E 6513722N | 81 | 58.4°N | 2021 | (-/-) |
| Agder | Nedre Jegersbergvann | Kristiansand | 88552E 6468627N | 29 | 58.1°N | 2022 | (M/-) |
| Agder | Nelaug* | Froland/Åmli | 128537E 6521603N | 141 | 58.7°N | 2013 | (M/B) |
| Agder | Nordvannet | Arendal | 123712E 6497127N | 124 | 58.5°N | 2014 | (-/-) |
| Agder | Ogge/Oggevatn | Birkenes/Iveland | 96560E 6496768N | 192 | 58.4°N | 1997 | (G/B) |
| Agder | River Fedaelva | Kvinesdal/Flekkefjord | 20987E 6488557N | 29 | 58.3°N | 2014 | (-/-) |
| Agder | River Honnemyrbekken | Vennesla | 90283E 6482403N | 127 | 58.2°N | 2011 | (-/-) |
| Agder | River Otra | Evje/Hornes/Bygland | 80889E 6512611N | 171 | 58.3°N | 2020 | (-/-) |
| Agder | River Sogndalselva/Søgneelva | Kristiansand | 78208E 6463462N | 7 | 58.1°N | 2022 | (-/-) |
| Agder | Selura | Flekkefjord | 14595E 6493024N | 32 | 58.3°N | 2019 | (G/-) |
| Agder | Skagestadsvatnet | Lindesnes | 63579E 6460495N | 3 | 58.0°N | 2021 | (M/-) |
| Agder | Stemmetjenn/Stemtjern | Arendal | 122544E 6498958N | 120 | 58.3°N | 2014 | (-/-) |
| Agder | Stortjern | Tvedestrand | 139395E 6514279N | 168 | 58.4°N | 2021 | (-/-) |
| Agder | Syndle | Grimstad | 121043E 6488985N | 39 | 58.2°N | 2023 | (G/B) |
| Agder | Sørsvann | Arendal | 133114E 6497041N | 39 | 58.5°N | 1997 | (-/-) |
| Agder | 3. stampe* | Kristiansand | 87698E 6467259N | 44 | 58.2°N | 2014 | (-/-) |
| Agder | Tretjønn* | Kristiansand | 90237E 6469179N | 72 | 58.2°N | 1983 | (-/-) |
| Agder | Trevann | Froland | 126671E 6501716N | 41 | 58.3°N | 2022 | (M/B) |
| Agder | Vollevannet | Kristiansand | 90509E 6468642N | 34 | 58.2°N | 2019 | (M/G) |
| Agder | Øksendalstjønna | Sirdal | 19745E 6524602N | 98 | 58.6°N | 2017 | (-/-) |
| Agder | Øvre Jegersbergvann | Kristiansand | 88885E 6469156N | 43 | 58.1°N | 2022 | (G/-) |
| Akershus | Bogstadvannet* ^π !^ | Bærum (Oslo) | 255483E 6656372N | 145 | 60.0°N | 1982 | (G/-) |
| Akershus | Burudvann | Bærum | 249718E 6657082N | 222 | 59.6°N | 2020 | (G/-) |
| Akershus | Dikemark-Verkensvannet | Asker | 240217E 6638932N | 181 | 59.8°N | 2014 | (M/B) |
| Akershus | Elgsjøen | Lunner | 259510E 6692674N | 341 | 60.3°N | 1995 | (M/-) |
| Akershus | Finnsrudvannet | Asker | 241434E 6641588N | 175 | 59.8°N | 2012 | (M/G) |
| Akershus | Flaskebekktjernet | Nesodden | 256707E 6642210N | 97 | 59.8°N | 2018 | (-/-) |
| Akershus | Fløyta | Lørenskog | 276023E 6643440N | 173 | 59.9°N | 2014 | (M/-) |
| Akershus | Gjellumvannet | Asker | 244176E 6637198N | 98 | 59.8°N | 2018 | (M/G) |
| Akershus | Gjersjøen | Nordre Follo/Ås | 263252E 6634995N | 40 | 59.8°N | 2018 | (M/G) |
| Akershus | Harestuvatnet* | Lunner | 262453E 6679943N | 235 | 60.2°N | 1992 | (M/-) |
| Akershus | Hurdalsjøen* | Eidsvoll/Hurdal | 281551E 6696736N | 175 | 60.3°N | 2014 | (G/-) |
| Akershus | Hvalstjern | Lillestrøm | 289666E 6647551N | 186 | 59.9°N | 2012 | (G/-) |
| Akershus | Hvamsetertjernet | Nes | 295465E 6667261N | 182 | 60.1°N | 2022 | (-/-) |
| Akershus | Høltjernet | Enebakk | 275565E 6627253.N | 137 | 59.7°N | 2012 | (-/-) |
| Akershus | Kjulstjernet | Nittedal | 269345E 6659745N | 350 | 60.0°N | 2020 | (-/-) |
| Akershus | Langvann | Nittedal (Oslo) | 264318E 6670812N | 342 | 60.1°N | 2016 | (-/-) |
| Akershus | Langvannet* | Lørenskog | 274069E 6651473N | 154 | 59.9°N | 2017 | (M/G) |
| Akershus | Ljøgodttjernet | Ullensaker | 285649E 6674179N | 185 | 60.1°N | 2014 | (-/-) |
| Akershus | Lyseren | Enebakk (Indre Østfold i Østfold) | 280413E 6623327N | 161 | 59.7°N | 2018 | (G/B) |
| Akershus | Midtsjøvannet | Nordre Follo | 268570E 6628609N | 129 | 59.7°N | 2014 | (M/-) |
| Akershus | Mjermen | Aurskog-Høland | 310389E 6626136N | 165 | 59.7°N | 2018 | (G/-) |
| Akershus | Mjøsa* ^π^ | Eidsvoll (Innlandet) | 283989E 6746369N | 121 | 60.7°N | 1994 | (G/B) |
| Akershus | Mylla | Lunner | 254624E 6686679N | 496 | 60.2°N | 2007 | (M/G) |
| Akershus | Myrdammen | Rælingen | 280454E 6642589N | 195 | 59.9°N | 2019 | (-/-) |
| Akershus | Mønevannet | Lørenskog | 275800E 6644102N | 169 | 59.5°N | 2022 | (M/-) |
| Akershus | Nordbytjern* | Ullensaker | 287203E 6675049N | 189 | 60.2°N | 2014 | (B/G) |
| Akershus | Nordvannet | Asker | 240374E 6639839N | 181 | 59.5°N | 2020 | (G/-) |
| Akershus | Pollevannet | Ås | 261342E 6630135N | 1 | 59.4°N | 2022 | (G/-) |
| Akershus | Randsfjorden* | Jevnaker/Gran/Søndre- og Nordre Land | 245214E 6687639N | 135 | 60.6°N | 1982 | (M/B) |
| Akershus | River Andelva | Eidsvoll | 291796E 6691675N | 175 | 60.3°N | 2014 | (-/-) |
| Akershus | River Glomma | Nes | 302463E 6668721N | 124 | 60.1°N | 2014 | (-/-) |
| Akershus | River Leira | Nannestad | 277449E 6681880N | 290 | 60.3°N | 2008 | (-/-) |
| Akershus | River Øverlandselva | Bærum | 252175E 6651836N | 76 | 59.6°N | 2023 | (-/-) |
| Akershus | Rognlivann | Bærum | 248738E 6660381N | 377 | 60.0°N | 2021 | (-/-) |
| Akershus | Romstjern | Nittedal | 270823E 6657114N | 267 | 59.6°N | 2023 | (G/-) |
| Akershus | Semsvannet* | Asker | 243685E 6644521N | 145 | 59.9°N | 1994 | (M/-) |
| Akershus | Setertjern | Nordre Follo (Oslo) | 270151E 6639014N | 136 | 59.5°N | 2022 | (G/-) |
| Akershus | Setten | Aurskog-Høland | 313813E 6637234N | 167 | 59.8°N | 2018 | (G/-) |
| Akershus | Skrukkelisjøen | Hurdal | 273532E 6706388N | 330 | 60.4°N | 1985 | (G/-) |
| Akershus | Stampetjernet | Lillestrøm | 285054E 6656263N | 161 | 59.6°N | 2023 | (-/-) |
| Akershus | Storetjernet | Jevnaker | 252135E 6690381N | 384 | 60.3°N | 2015 | (-/-) |
| Akershus | Storøyungen | Nittedal/Nannestad | 272099E 6675986N | 331 | 60.2°N | 2009 | (G/-) |
| Akershus | Svartputt* | Asker | 241274E 6640834N | 207 | 59.8°N | 2008 | (-/-) |
| Akershus | Svea/Sveavann | Lunner | 256845E 6687787N | 481 | 60.3°N | 1994 | (M/-) |
| Akershus | Syverrudtjern | Nordre Follo | 270621E 6636461N | 152 | 59.8°N | 2019 | (-/-) |
| Akershus | Søndre Movann | Nittedal (Oslo) | 266901E 6662890N | 273 | 60.0°N | 2012 | (-/-) |
| Akershus | Tussetjern | Nordre Follo | 265784E 6632370N | 94 | 59.8°N | 2018 | (M/G) |
| Akershus | Utsjøen | Eidsvoll/Nes | 301986E 6694640N | 268 | 60.2°N | 2020 | (G/-) |
| Akershus | Varsjøen | Lillestrøm | 290516E 6651023N | 192 | 59.6°N | 2020 | (G/G) |
| Akershus | Vesledammen | Asker | 241672E 6633839N | 188 | 59.5°N | 2022 | (-/-) |
| Akershus | Vesletjern* | Lørenskog | 274775E 6650990N | 155 | 59.9°N | 2010 | (-/-) |
| Akershus | Vientjern | Nordre Follo (Indre Østfold i Østfold) | 272499E 6623490N | 183 | 59.7°N | 2018 | (-/-) |
| Akershus | Våg/Vågvann | Enebakk | 276294E 6628276N | 126 | 59.7°N | 2017 | (M/-) |
| Akershus | Østernvann | Bærum | 253455E 6656162N | 215 | 60.0°N | 2018 | (G/-) |
| Buskerud | Andedammen | Drammen | 230289E 6631459N | 14 | 59.7°N | 2007 | (-/-) |
| Buskerud | Bergsjøen | Modum | 220010E 6655891N | 61 | 59.9°N | 2018 | (G/G) |
| Buskerud | Damtjern* | Lier | 235619E 6638931N | 194 | 59.8°N | 1992 | (M/G) |
| Buskerud | Eikdammen | Drammen | 239769E 6617405N | 99 | 59.8°N | 2013 | (-/-) |
| Buskerud | Eikeren* ^π^ | Øvre Eiker (Holmestrand i Vestfold) | 214166E 6624625N | 19 | 59.6°N | 1982 | (M/B) |
| Buskerud | Fiskumvannet* | Øvre Eiker | 209215E 6630089N | 19 | 59.7°N | 1982 | (M/-) |
| Buskerud | Grytevatnet | Hemsedal | 160801E 6761309N | 830 | 60.8°N | 2017 | (-/-) |
| Buskerud | Hajeren | Kongsberg (Holmestrand i Vestfold) | 213825E 6613759N | 411 | 59.6°N | 2012 | (M/-) |
| Buskerud | Helsingvatnet | Hemsedal | 158237E 6763092N | 829 | 60.9°N | 2013 | (M/-) |
| Buskerud | Krøderen* | Flå/Krødsherad | 205935E 6681130N | 133 | 60.2°N | 2014 | (M/B) |
| Buskerud | Landfalltjernet | Drammen | 228537E 6635671N | 315 | 59.8°N | 2014 | (M/-) |
| Buskerud | Lauvnesvatnet | Sigdal/Flesberg | 197349E 6660535N | 367 | 60.0°N | 2019 | (G/-) |
| Buskerud | Mysutjernet | Kongsberg | 197886E 6614163N | 336 | 59.6°N | 2019 | (-/-) |
| Buskerud | Nerdammen | Drammen | 223969E 6639431N | 435 | 59.5°N | 2023 | (G/-) |
| Buskerud | Plassedammen | Hole | 243574E 6664876N | 271 | 60.0°N | 2014 | (G/-) |
| Buskerud | River Bingselva | Øvre Eiker | 213066E 6644676N | 40 | 59.8°N | 2015 | (-/-) |
| Buskerud | River Drammenselva | Drammen | 229522E 6632951N | 6 | 59.8°N | 1992 | (-/-) |
| Buskerud | River Kobberbergselva | Kongsberg | 195617E 6622327N | 186 | 59.4°N | 2022 | (-/-) |
| Buskerud | River Randselva | Ringerike | 240971E 6682464N | 95 | 60.1°N | 2021 | (-/-) |
| Buskerud | River Storelva | Sigdal | 192607E 6673943N | 175 | 60.1°N | 2014 | (-/-) |
| Buskerud | Soneren | Sigdal | 194763E 6671167N | 104 | 60.1°N | 2014 | (G/G) |
| Buskerud | Sperillen* | Ringerike | 228800E 6710582N | 150 | 60.4°N | 1982 | (M/B) |
| Buskerud | Steinsfjorden* | Ringerike/Hole | 240818E 6673926N | 63 | 60.1°N | 1982 | (M/B) |
| Buskerud | Stor/Konneruddammen * ^π^ | Drammen | 225918E 6630502N | 245 | 59.7°N | 1989 | (M/-) |
| Buskerud | Storevatnet | Hemsedal | 161867E 6763634N | 824 | 60.9°N | 2017 | (M/-) |
| Buskerud | Strandafjorden* | Ål | 143093E 6735452N | 445 | 60.6°N | 2011 | (G/-) |
| Buskerud | Stærnestjønn | Rollag | 182067E 6662569N | 199 | 60.0°N | 1982 | (-/-) |
| Buskerud | Sundtjønn | Rollag | 169410E 6665010N | 816 | 59.6°N | 2023 | (-/-) |
| Buskerud | Torevatnet | Ringerike | 216381E 6689060N | 143 | 60.2°N | 2013 | (G/-) |
| Buskerud | Trytetjern | Nesbyen | 179932E 6729728N | 509 | 60.3°N | 2020 | (-/-) |
| Buskerud | Tyrifjorden* ^π^ | Ringerike/Hole/Lier/Modum | 235062E 6663017N | 63 | 60.0°N | 1982 | (G/B) |
| Buskerud | Vannverksdammen | Drammen | 226801E 6631936N | 189 | 59.4°N | 2023 | (-/-) |
| Buskerud | Øksne | Kongsberg | 188751E 6619420N | 389 | 59.4°N | 2023 | (M/-) |
| Buskerud | Årbogen | Drammen | 219168E 6636825N | 189 | 59.8°N | 2014 | (-/-) |
| Finnmark | Áidneluoppal | Nesseby | 1013176E 7824767N | 160 | 70.0°N | 2020 | (VG/-) |
| Finnmark | Nedre Neverfjordvatnet | Hammerfest | 829343E 7838279N | 128 | 70.4°N | 2014 | (VG/-) |
| Finnmark | Ørnevatnet | Sør-Varanger | 1078777E 7791596N | 72 | 69.6°N | 2014 | (VG/B) |
| Innlandet | Aurdalsfjorden* | Nord-Aurdal | 194136E 6768699N | 307 | 60.9°N | 1982 | (G/-) |
| Innlandet | Baksjøen | Åsnes | 336202E 6728550N | 196 | 60.4°N | 2023 | (G/-) |
| Innlandet | Bergsjøen | Stange | 299498E 6716972N | 363 | 60.5°N | 2011 | (G/-) |
| Innlandet | Bekkholtjønna | Folldal | 248453E 6899089N | 666 | 62.1°N | 2021 | (-/-) |
| Innlandet | Bæreia | Kongsvinger | 331670E 6673048N | 232 | 60.1°N | 2021 | (M/-) |
| Innlandet | Båtstøtjønnet | Øyer | 248154E 6807737N | 180 | 61.3°N | 2014 | (-/-) |
| Innlandet | Drevsjøen | Engerdal | 343056E 6865534N | 670 | 61.9°N | 2014 | (M/-) |
| Innlandet | Einavatnet* | Vestre Toten | 260747E 6723074N | 398 | 60.6°N | 2007 | (B/G) |
| Innlandet | Fjorda/Velmunden | Gran | 241285E 6711519N | 389 | 60.4°N | 2011 | (G/-) |
| Innlandet | Flatningen | Vågå | 190221E 6865936N | 776 | 61.8°N | 2018 | (G/-) |
| Innlandet | Flensjøen* | Trysil | 354840E 6816481N | 641 | 61.5°N | 1982 | (M/-) |
| Innlandet | Fløafjorden | Nord-Aurdal | 191916E 6770972N | 318 | 61.0°N | 1982 | (G/-) |
| Innlandet | Gålåvatnet | Sør-Fron | 221197E 6831396N | 778 | 61.3°N | 2021 | (VG/-) |
| Innlandet | Harasjøen | Stange | 304638E 6727598N | 280 | 60.6°N | 2014 | (G/G) |
| Innlandet | Harsjøen/Harrsjøen | Rendalen | 290844E 6864408N | 676 | 60.1°N | 2014 | (G/-) |
| Innlandet | Hemsjøen* | Åmot | 307230E 6778950N | 352 | 61.1°N | 1996 | (-/-) |
| Innlandet | Helgesjøen | Eidskog | 334361E 6643857N | 163 | 59.9°N | 2019 | (M/-) |
| Innlandet | Hersjøen | Østre Toten | 282486E 6717396N | 491 | 60.3°N | 2020 | (G/G) |
| Innlandet | Høversjøen | Østre Toten | 275048E 6714866N | 346 | 60.5°N | 2018 | (G/-) |
| Innlandet | Kroktjønna | Folldal | 248622E 6899389N | 667 | 62.1°N | 2021 | (-/-) |
| Innlandet | Leirin | Nord-Aurdal | 191850E 6778505N | 818 | 61.0°N | 2017 | (G/B) |
| Innlandet | Lesjaskogsvatnet | Lesja | 159619E 6915046N | 611 | 62.2°N | 2017 | (G/-) |
| Innlandet | Lierdammen | Kongsvinger | 336675E 6670002N | 183 | 60.1°N | 2014 | (-/-) |
| Innlandet | Lygna | Gran | 260723E 6709956N | 620 | 60.5°N | 2014 | (-/-) |
| Innlandet | Mesnadammen | Lillehammer | 258577E 6784574N | 480 | 61.1°N | 2020 | (-/-) |
| Innlandet | Mjøsa* ^π^ | Hamar/Ringsaker/Gjøvik/Lillehammer | 283989E 6746369N | 121 | 60.7°N | 1994 | (G/B) |
| Innlandet | Nessjøen | Eidskog | 344679E 6653153N | 132 | 59.6°N | 2021 | (M/-) |
| Innlandet | Nordersjøen | Tolga | 301626E 6919759N | 764 | 62.2°N | 2021 | (G/-) |
| Innlandet | Nord-Mesna | Ringsaker | 265004E 6783279N | 520 | 61.1°N | 1986 | (G/-) |
| Innlandet | Næra | Ringsaker | 268285E 6772164N | 339 | 61.0°N | 2013 | (G/G) |
| Innlandet | Nørdre Syndin | Vestre Slidre/Vang | 160310E 6789419N | 937 | 61.1°N | 2021 | (G/-) |
| Innlandet | Randsfjorden* | Jevnaker/Gran/Søndre- og Nordre Land | 245214E 6687639N | 135 | 60.6°N | 1982 | (M/B) |
| Innlandet | Reinsvatnet | Lillehammer/Øyer | 264536E 6794246N | 906 | 61.1°N | 2020 | (B/-) |
| Innlandet | River Flasåne | Øystre Slidre | 191910E 6787245N | 927 | 61.1°N | 2020 | (-/-) |
| Innlandet | River Flisa | Åsnes | 346173E 6728759N | 201 | 60.4°N | 2021 | (-/-) |
| Innlandet | River Gausa | Lillehammer | 251277E 6789220N | 130 | 61.2°N | 2013 | (-/-) |
| Innlandet | River Gudbrandsdalslågen | Lillehammer | 253996E 6785621N | 126 | 61.1°N | 2020 | (-/-) |
| Innlandet | River Lenaelva | Østre Toten | 267770E 6726373N | 182 | 60.6°N | 2018 | (-/-) |
| Innlandet | River Rena* | Rena | 305629E 6783404N | 216 | 61.1°N | 1982 | (-/-) |
| Innlandet | River Sjøåa | Trysil | 339123E 6819112 N | 594 | 61.5°N | 2019 | (-/-) |
| Innlandet | River Stokkelva | Gjøvik | 265256E 6757950N | 542 | 60.9°N | 2013 | (-/-) |
| Innlandet | Rokosjøen* | Løten/Elverum | 306218E 6744380N | 217 | 60.8°N | 2013 | (M/-) |
| Innlandet | Sagtjernet | Elverum | 313967E 6755995N | 181 | 60.5°N | 2020 | (M/-) |
| Innlandet | Savalen | Tynset/Alvdal | 264392E 6909630N | 707 | 62.1°N | 2023 | (M/G) |
| Innlandet | Setningen | Stor-Elvdal | 249052E 6858494N | 757 | 61.8°N | 2012 | (VG/G) |
| Innlandet | Siap (artificial pond) | Sør-Odal | 317445E 6685509N | 138 | 60.3°N | 2017 | (-/-) |
| Innlandet | Sigernessjøen | Kongsvinger | 335575E 6668756N | 184 | 60.1°N | 2019 | (G/-) |
| Innlandet | Sillungen/Sillongen | Vestre Toten | 266794E 6736755N | 452 | 60.7°N | 2011 | (M/-) |
| Innlandet | Sjusjøen* | Ringsaker | 269173E 6786717N | 810 | 61.2°N | 2012 | (B/G) |
| Innlandet | Skasen | Kongsvinger/Grue | 352438E 6697441N | 265 | 60.4°N | 2014 | (G/G) |
| Innlandet | Skonnolstjernet | Gjøvik | 250728E 6757972N | 433 | 60.5°N | 2022 | (-/-) |
| Innlandet | Stavåsdammen | Elverum | 315047E 6758749N | 294 | 60.6°N | 2021 | (VG/-) |
| Innlandet | Steffenrudstjernet | Vestre Toten | 264804E 6734787N | 413 | 60.7°N | 2018 | (M/-) |
| Innlandet | Storsjøen | Nord-Odal/Sør-Odal | 315180E 6697019N | 131 | 60.2°N | 2021 | (G/G) |
| Innlandet | Surtjennet | Elverum | 320484E 6746146N | 183 | 60.5°N | 2023 | (-/-) |
| Innlandet | Svarttjønna | Tynset | 359611E 6802176N | 583 | 62.2°N | 2014 | (-/-) |
| Innlandet | Synnfjorden | Nordre Land | 223470E 6787744N | 796 | 61.1°N | 2012 | (G/-) |
| Innlandet | Sør-Mesna | Ringsaker | 270316E 6779848N | 522 | 61.1°N | 1992 | (G/-) |
| Innlandet | Tjønna* | Trysil | 355959E 6795644N | 359 | 61.3°N | 2009 | (-/-) |
| Innlandet | Trevatna | Søndre Land | 249411E 6732667N | 385 | 60.7°N | 2011 | (M/-) |
| Innlandet | Utgardsjøen | Kongsvinger | 354221E 6656745N | 127 | 60.0°N | 2011 | (G/-) |
| Innlandet | Vassbråa | Gran | 265202E 6701235N | 547 | 60.4°N | 2017 | (G/-) |
| Innlandet | Vermunden | Åsnes | 356821E 6732190N | 215 | 60.4°N | 2020 | (M/-) |
| Innlandet | Vesletjernet | Lillehammer | 250195E 6780964N | 535 | 61.1°N | 2023 | (-/-) |
| Innlandet | Vingersjøen | Kongsvinger | 336633E 6674895N | 143 | 60.2°N | 2005 | (M/-) |
| Innlandet | Volbufjorden | Øystre Slidre | 181339E 6788449N | 434 | 61.1°N | 2021 | (G/-) |
| Innlandet | Åbortjernet | Gjøvik | 246714E 6762487N | 576 | 60.5°N | 2020 | (-/-) |
| Møre og Romsdal | Brusdalsvatnet | Ålesund | 61239E 6956393N | 26 | 62.5°N | 2008 | (VG/G) |
| Møre og Romsdal | Mausavatnet | Sula | 49663E 6950425N | 46 | 62.4°N | 2019 | (G/-) |
| Møre og Romsdal | Osvatnet | Molde | 131972E 6983989N | 12 | 62.8°N | 2019 | (G/-) |
| Møre og Romsdal | Storvatnet | Averøy | 126644E 7012200N | 26 | 63.0°N | 2023 | (G/-) |
| Møre og Romsdal | Øverlandsvatnet | Molde | 102592E 6982857N | 245 | 62.5°N | 2023 | (G/-) |
| Nordland | Bleiksvatnet | Andøy | 533004E 7643908N | 28 | 69.3°N | 2004 | (VG/G) |
| Nordland | Brennvikvatnet | Hamarøy | 527566E 7556283N | 13 | 68.1°N | 2020 | (G/-) |
| Nordland | Djupvatnet | Narvik | 568721E 7577102N | 77 | 68.3°N | 2014 | (-/-) |
| Nordland | Fjærvatnet | Bodø | 490204E 7486255N | 6 | 67.5°N | 2004 | (G/-) |
| Nordland | Forsa-litlvatnet | Hamarøy | 544326E 7559586N | 92 | 68.2°N | 2012 | (-/-) |
| Nordland | Forsavatnet | Narvik | 567384E 7575737N | 30 | 68.3°N | 2012 | (G/-) |
| Nordland | Fustvatnet | Vefsn | 426277E 7310243N | 38 | 65.5°N | 2021 | (M/-) |
| Nordland | Gangvatnet | Bodø | 475931E 7456188N | 37 | 67.2°N | 2018 | (-/-) |
| Nordland | Grunnvatnet | Andøy | 528976E 7663833N | 10 | 69.1°N | 2011 | (G/-) |
| Nordland | Holmvatnet* | Vega | 356531E 7287400N | 28 | 65.7°N | 1998 | (-/-) |
| Nordland | Horndalsvatnet | Sørfold | 530168E 7502667N | 112 | 67.4°N | 2023 | (VG/-) |
| Nordland | Lilandsvatnet/Rystadvatnet | Vestvågøy | 448459E 7569297N | 13 | 68.2°N | 2014 | (M/G) |
| Nordland | Markvatnet | Meløy | 445772E 7421488N | 26 | 66.9°N | 2004 | (G/-) |
| Nordland | Steinslandvannet/Nyvollvannet | Steigen | 510621E 7528617N | 20 | 67.9°N | 2006 | (-/-) |
| Nordland | Osvatnet/Osmarkvatnet | Evenes | 575080E 7599440N | 44 | 68.5°N | 2014 | (-/-) |
| Nordland | Pollåsvatnet | Bø | 483540E 7616347N | 13 | 68.4°N | 2022 | (G/-) |
| Nordland | Rauvatnet* | Rana | 478626E 7350952N | 488 | 66.3°N | 2002 | (-/-) |
| Nordland | River Reipåga (Skjærhøla) | Meløy | 440544E 7421995N | 20 | 66.9°N | 2004 | (-/-) |
| Nordland | River Selneselva | Sortland | 515982E 7622264N | 13 | 68.4°N | 2023 | (-/-) |
| Nordland | River Åseelva | Andøy | 529620E 7656548N | 16 | 69.0°N | 2011 | (-/-) |
| Nordland | Skilvatnet | Hamarøy | 537324E 7550262N | 35 | 68.1°N | 2014 | (G/-) |
| Nordland | Soløyvatnet* ^π^ | Bodø | 482350E 7466518N | 49 | 67.3°N | 2003 | (G/-) |
| Nordland | Sommarvatnet | Evenes | 571183E 7601756N | 39 | 68.5°N | 2014 | (-/-) |
| Nordland | Storsvenningvatnet | Grane | 423377E 7245727N | 184 | 65.2°N | 2011 | (G/G) |
| Nordland | Storvatnet/Opsjøvatnet | Brønnøy | 389083E 7255991N | 5 | 65.4°N | 2011 | (-/-) |
| Nordland | Storvatnet | Flakstad | 432542E 7557355N | 10 | 68.1°N | 2015 | (G/-) |
| Nordland | Sørvågvatnet | Øksnes | 508278E 7644601N | 5 | 68.9°N | 2014 | (G/-) |
| Nordland | Tverråsvatnet | Hamarøy | 535557E 7550692N | 40 | 68.0°N | 2021 | (-/-) |
| Nordland | Vatnvatnet | Bodø | 490742E 7468725N | 4 | 67.3°N | 2004 | (M/-) |
| Nordland | Vågøyvatnet | Bodø | 478522E 7465274N | 120 | 67.3°N | 2011 | (G/-) |
| Nordland | Ømmervatnet* | Vefsn | 427528E 7319843N | 42 | 66.0°N | 1994 | (-/-) |
| Oslo | Bogstadvannet* ^π !^ | Oslo (Bærum i Akershus) | 255483E 6656372N | 145 | 60.0°N | 1982 | (G/-) |
| Oslo | Bjørnsjøen | Oslo | 258895E 6667383N | 337 | 60.1°N | 2014 | (G/B) |
| Oslo | Båntjern | Oslo | 260359E 6655274N | 248 | 59.6°N | 2023 | (-/-) |
| Oslo | Dronningdammen | Oslo | 261063E 6649934N | 31 | 59.6°N | 2022 | (-/-) |
| Oslo | Groruddammen/Badedammen* | Oslo | 269802E 6653912N | 167 | 60.0°N | 1996 | (G/-) |
| Oslo | Isdammen | Oslo | 266845E 6653861N | 232 | 59.6°N | 2023 | (-/-) |
| Oslo | Langvann | Oslo (Nittedal i Akershus) | 264318E 6670812N | 342 | 60.1°N | 2016 | (-/-) |
| Oslo | Lauvtjern | Oslo | 270565E 6648700N | 342 | 59.9°N | 2017 | (-/-) |
| Oslo | Lillevann | Oslo | 257700E 6657242N | 423 | 60.0°N | 1989 | (-/-) |
| Oslo | Lutvann* ^π^ | Oslo | 269571E 6648951N | 205 | 59.9°N | 2002 | (VG/-) |
| Oslo | Middelalderdammen | Oslo | 263013E 6648377N | 3 | 59.9°N | 2002 | (-/-) |
| Oslo | Midtstudammen | Oslo | 258923E 6655411N | 258 | 60.0°N | 2017 | (-/-) |
| Oslo | Nøklevann* | Oslo | 269277E 6644791N | 163 | 59.9°N | 2004 | (VG/-) |
| Oslo | River Akerselva | Oslo | 264353E 6655228N | 144 | 60.0°N | 2014 | (-/-) |
| Oslo | Rottungen | Oslo | 261462E 6664577N | 310 | 60.0°N | 2012 | (G/-) |
| Oslo | Setertjern | Oslo (Nordre Follo i Akershus) | 270151E 6639014N | 136 | 59.5°N | 2022 | (G/-) |
| Oslo | Skraperudtjern* | Oslo | 267778E 6644130N | 119 | 59.9°N | 1982 | (-/-) |
| Oslo | Sognsvann* ^π^ | Oslo | 261699E 6656321N | 183 | 60.0°N | 1982 | (G/-) |
| Oslo | Steinbruvann | Oslo | 270281E 6656044N | 257 | 60.0°N | 2018 | (G/-) |
| Oslo | Stensrudtjern | Oslo | 268556E 6638752N | 133 | 59.5°N | 2020 | (-/-) |
| Oslo | Store Åklungen | Oslo | 261444E 6659297N | 295 | 60.0°N | 2013 | (G/-) |
| Oslo | Søndre Movann | Oslo (Nittedal i Akershus) | 266901E 6662890N | 273 | 60.0°N | 2012 | (-/-) |
| Oslo | Svartkulp* | Oslo | 269798E 6641217N | 247 | 60.0°N | 2012 | (G/-) |
| Oslo | Trollvann | Oslo | 266031E 6654518N | 311 | 60.0°N | 2018 | (G/-) |
| Oslo | Ulsrudvann* | Oslo | 268684E 6646182N | 188 | 59.9°N | 1992 | (-/-) |
| Oslo | Vesletjern/Lilletjern* | Oslo | 268946E 6654144N | 231 | 60.0°N | 2008 | (G/G) |
| Oslo | Øvresetertjern | Oslo | 258572E 6657392N | 478 | 59.6°N | 2021 | (G/-) |
| Oslo | Øyungen | Oslo | 263036E 6663643N | 283 | 60.0°N | 2019 | (VG/-) |
| Oslo | Årvolldammen* | Oslo | 266591E 6652956N | 181 | 59.9°N | 1997 | (-/-) |
| Rogaland | Aksdalsvatnet | Tysvær | -42541E 6624828N | 18 | 59.2°N | 2023 | (VG/-) |
| Rogaland | Bilstadvatnet | Lund | -3373E 6521440N | 183 | 58.5°N | 2013 | (M/G) |
| Rogaland | Bjørheimsvatnet | Strand | -15987E 6582177N | 33 | 59.1°N | 2018 | (G/-) |
| Rogaland | Bongsatjørna | Tysvær | -41418E 6626048N | 25 | 59.3°N | 2023 | (M/B) |
| Rogaland | Botnavatnet | Tysvær | -33451E 6615171N | 5 | 59.2°N | 2022 | (-/-) |
| Rogaland | Bråsteinvatnet* | Sandnes | -31114E 6554864N | 46 | 58.4°N | 2010 | (M/-) |
| Rogaland | Edlandsvatnet | Gjesdal | -26937E 6549434N | 104 | 58.5°N | 2023 | (M/G) |
| Rogaland | Eikelivatnet | Hjelmeland | 6921E 6600550N | 306 | 58.9°N | 2013 | (-/-) |
| Rogaland | Eikesvatnet | Bjerkreim | -23197E 6522180N | 24 | 58.5°N | 1993 | (G/-) |
| Rogaland | Eivindsholttjørn | Time | -39656E 6547056N | 34 | 58.4°N | 2020 | (-/-) |
| Rogaland | Eivindsvatnet | Haugesund | -48784E 6626647N | 64 | 59.4°N | 2012 | (G/-) |
| Rogaland | Floen | Sandnes | -26959E 6560527N | 27 | 58.9°N | 2018 | (-/-) |
| Rogaland | Fjermestadvatnet | Gjesdal | -29592E 6549951N | 122 | 58.8°N | 2019 | (M/-) |
| Rogaland | Frøylandsvatnet* | Klepp/Time | -38705E 6549075N | 24 | 58.8°N | 2010 | (M/-) |
| Rogaland | Første vannbasseng | Eigersund | -23191E 6514097N | 72 | 58.5°N | 1992 | (-/-) |
| Rogaland | Gloppevatnet | Gjesdal | -13395E 6548436N | 256 | 58.8°N | 2017 | (-/-) |
| Rogaland | Grindavatnet/Nordavatnet | Tysvær | -39136E 6626467N | 27 | 59.3°N | 2022 | (-/-) |
| Rogaland | Hellvigsvatnet | Eigersund | -29849E 6519217N | 11 | 58.3°N | 2021 | (G/-) |
| Rogaland | Heståvatn | Sandnes | -25458E 6568768N | 15 | 58.9°N | 2018 | (-/-) |
| Rogaland | Horvetjørna | Sandnes | -21276E 6564592N | 23 | 58.5°N | 2020 | (-/-) |
| Rogaland | Iglatjørna | Sandnes | -23652E 6567300N | 27 | 58.9°N | 2019 | (-/-) |
| Rogaland | Langavatnet | Time | -31814E 6540754N | 154 | 58.4°N | 2023 | (-/-) |
| Rogaland | Langevatnet | Eigersund | -23003E 6516480N | 39 | 58.5°N | 2014 | (-/-) |
| Rogaland | Litla Fotvatnet | Karmøy | -52039E 6613242N | 14 | 59.2°N | 2023 | (-/-) |
| Rogaland | Lutsivatnet* ^π^ | Sandnes | -28125E 6561928N | 26 | 58.9°N | 2003 | (M/-) |
| Rogaland | Nordvatnet | Strand | -19434E 6583802N | 38 | 59.1°N | 2014 | (M/B) |
| Rogaland | Oltedalsvatnet | Gjesdal | -18086E 6554273N | 108 | 58.8°N | 2018 | (G/-) |
| Rogaland | Orrevatnet | Klepp | -46444E 6550559N | 4 | 58.5°N | 2023 | (B/-) |
| Rogaland | River Figgjo | Sandnes/Klepp/Time/Gjesdal | -33527E 6554846N | 24 | 58.5°N | 2023 | (-/-) |
| Rogaland | Sandtjødne/Sandtjørna | Eigersund | -23206E 6513560N | 92 | 58.5°N | 1992 | (-/-) |
| Rogaland | Skjelbreitjørna | Sandnes | -26097E 6557278N | 106 | 58.8°N | 2013 | (G/-) |
| Rogaland | Stemmen | Time | -35123E 6552535N | 31 | 58.7°N | 2017 | (M/-) |
| Rogaland | Stora Fotvatnet | Karmøy | -52556E 6613360N | 17 | 59.2°N | 2023 | (G/-) |
| Rogaland | Store Stokkavatn | Stavanger | -35577E 6573674N | 11 | 59.0°N | 2019 | (VG/-) |
| Rogaland | Stokkalandsvatnet | Sandnes | -34234E 6557175N | 18 | 58.8°N | 2003 | (M/G) |
| Rogaland | Svanevatnet | Eigersund | -22541E 6518005N | 52 | 58.5°N | 1993 | (-/-) |
| Rogaland | Vigdarvatnet | Haugesund (Sveio i Vestland) | -44039E 6634828N | 8 | 59.3°N | 2019 | (M/B) |
| Rogaland | Åsvatnet | Time | -33480E 6548254N | 157 | 58.4°N | 2020 | (G/-) |
| Telemark | Bjønntjønn | Nissedal | 135682E 6560871N | 416 | 59.0°N | 2014 | (-/-) |
| Telemark | Dalsvatn | Midt-Telemark | 179410E 6595444N | 166 | 59.4°N | 2016 | (G/-) |
| Telemark | Farris/Farrisvannet | Siljan (Larvik/Porsgrunn i Vestfold) | 213935E 6557687N | 23 | 59.2°N | 2012 | (G/G) |
| Telemark | Follsjå | Notodden | 173813E 6631330N | 225 | 59.4°N | 2023 | (G/-) |
| Telemark | Gorningen | Siljan | 202856E 6579293N | 75 | 59.2°N | 2023 | (G/-) |
| Telemark | Heivannet | Siljan | 196471E 6581358N | 237 | 59.2°N | 2021 | (G/-) |
| Telemark | Kilevann | Skien | 180161E 6568693N | 62 | 59.1°N | 2016 | (M/G) |
| Telemark | Kviteseidvatnet | Kviteseid | 134680E 6596966N | 72 | 59.4°N | 2017 | (G/-) |
| Telemark | Langen | Nome | 167417E 6579353N | 100 | 59.2°N | 2010 | (G/-) |
| Telemark | Lognvikvatn | Vinje | 114287E 6635015N | 697 | 59.7°N | 2018 | (M/G) |
| Telemark | Morgedalstjønni | Kviteseid | 127088E 6612276N | 423 | 59.5°N | 2018 | (M/-) |
| Telemark | Nisser | Nissedal/Kviteseid | 126891E 6583427N | 247 | 59.1°N | 2013 | (G/B) |
| Telemark | Nomevatn* | Nome | 168440E 6587007N | 59 | 59.3°N | 2011 | (-/-) |
| Telemark | Norsjø* ^π^ | Skien/Nome/Midt-Telemark | 173099E 6596288N | 15 | 59.3°N | 2009 | (M/B) |
| Telemark | Nossevatn | Tokke | 114211E 6612501N | 555 | 59.5°N | 2012 | (-/-) |
| Telemark | River Eidselva | Nome | 172375E 6585597N | 27 | 59.2°N | 2022 | (-/-) |
| Telemark | River Gvarvelva | Midt-Telemark | 169733E 6597463N | 16 | 59.2°N | 2021 | (-/-) |
| Telemark | River Lundereidelva | Drangedal | 172271E 6551470N | 60 | 58.6°N | 2023 | (-/-) |
| Telemark | River Sauarelva | Midt-Telemark | 175855E 6599186N | 26 | 59.4°N | 1995 | (-/-) |
| Telemark | River Skienselva/Porsgrunnselva | Porsgrunn | 194058E 6567945N | 1 | 59.2°N | 2001 | (-/-) |
| Telemark | River Straumen | Nome | 164673E 6588622N | 63 | 59.3°N | 2017 | (-/-) |
| Telemark | Sannervannet | Nome | 169525E 6576630N | 99 | 59.1°N | 2020 | (G/-) |
| Telemark | Seljordsvatn* | Seljord | 143756E 6607426N | 116 | 59.4°N | 2009 | (VG/-) |
| Telemark | Sundkilen | Kviteseid | 130734E 6601821N | 72 | 59.4°N | 2012 | (G/-) |
| Telemark | Toke/Tokke/Tokevann* | Bamble/Drangedal/Kragerø | 167328E 6552494N | 61 | 59.1°N | 1994 | (G/-) |
| Telemark | Tyrivatn | Nome | 166246E 6583697N | 96 | 59.3°N | 2010 | (G/-) |
| Telemark | Øverbøtjønn* | Siljan | 200529E 6583187N | 96 | 59.3°N | 1983 | (-/-) |
| Telemark | Åletjern | Skien | 188227E 6578107N | 99 | 59.2°N | 2016 | (-/-) |
| Troms | Anemyrvatnet | Ibestad | 579407E 7628152N | 178 | 68.8°N | 2014 | (-/-) |
| Troms | Finnfjordvatn | Målselv/Sørreisa/Senja | 626734E 7682578N | 25 | 69.1°N | 1991 | (VG/-) |
| Troms | Grunnvatnet | Harstad | 560233E 7631962N | 98 | 68.5°N | 2020 | (-/-) |
| Troms | Litle Trøsevatnet | Tjeldsund | 570520E 7607631N | 119 | 68.6°N | 2008 | (-/-) |
| Troms | Musvatnet | Harstad | 559106E 7629226N | 320 | 68.5°N | 2022 | (-/-) |
| Troms | Møkkelandsvatnet | Harstad | 557485E 7634863N | 13 | 68.5°N | 2023 | (VG/-) |
| Troms | River Badderelva | Kvænangen | 775228E 7762243N | 39 | 69.5°N | 2022 | (-/-) |
| Troms | Røyrbakkvatnet | Salangen | 609883E 7653013N | 26 | 68.9°N | 2019 | (G/-) |
| Troms | Steinsåsvatnet | Ibestad | 580460 E 7632165N | 132 | 68.8°N | 2016 | (-/-) |
| Troms | Storvatnet | Senja | 615584E 7684817N | 31 | 69.1°N | 2023 | (VG/B) |
| Troms | Sørlivatnet | Senja | 604472E 7684820N | 15 | 69.3°N | 2015 | (VB/-) |
| Troms | Vikevatnet | Harstad | 561692E 7618342N | 31 | 68.4°N | 2023 | (G/-) |
| Troms | Abborvatn/Åbbortjern | Bardu | 639436E 7638435N | 72 | 68.5°N | 2009 | (-/-) |
| Trøndelag | Almovatnet | Stjørdal | 301923E 7049822N | 136 | 63.5°N | 2011 | (G/-) |
| Trøndelag | Aunvatnet | Frøya | 185005E 7081185N | 20 | 63.4°N | 2022 | (-/-) |
| Trøndelag | Ausetvatnet | Stjørdal | 305644E 7052586N | 201 | 63.5°N | 2017 | (G/-) |
| Trøndelag | Baklidammen | Trondheim | 266556E 7040832N | 196 | 63.3°N | 2023 | (M/-) |
| Trøndelag | Byavatnet* | Levanger | 303099E 7065699N | 40 | 63.7°N | 2008 | (VG/-) |
| Trøndelag | Estenstaddammen | Trondheim | 274639E 7037107N | 248 | 63.4°N | 2015 | (G/-) |
| Trøndelag | Feragen | Røros | 339355E 6932721N | 655 | 62.5°N | 2018 | (G/-) |
| Trøndelag | Flaksjøen* | Stjørdal | 307240E 7038716N | 294 | 63.4°N | 2011 | (-/-) |
| Trøndelag | Frilsjøen* | Orkland | 234766E 7005996N | 349 | 63.1°N | 2014 | (VG/-) |
| Trøndelag | Gangåsvatnet | Orkland | 231088E 7027423N | 153 | 63.3°N | 2014 | (M/G) |
| Trøndelag | Grønlivatnet | Rindal | 212195E 7006515N | 281 | 63.1°N | 2005 | (G/-) |
| Trøndelag | Grønsjøen | Levanger | 300418E 7067403N | 142 | 63.8°N | 1982 | (-/-) |
| Trøndelag | Gaustadvatnet | Melhus | 253518E 7011583N | 164 | 63.1°N | 2021 | (G/-) |
| Trøndelag | Gjøljavatnet | Ørland | 252961E 7084126N | 52 | 63.8°N | 2015 | (M/-) |
| Trøndelag | Haukvatnet | Trondheim | 266197E 7037903N | 189 | 63.4°N | 2017 | (-/-) |
| Trøndelag | Hammervatnet* | Levanger | 301957E 7060171N | 24 | 63.6°N | 1982 | (M/G) |
| Trøndelag | Heimsvatnet* | Heim | 203523E 7045972N | 45 | 63.4°N | 2004 | (M/G) |
| Trøndelag | Hessjøen/Hestsjøen | Stjørdal | 302515E 7046908N | 177 | 63.5°N | 2016 | (-/-) |
| Trøndelag | Hestsjøen* | Trondheim | 280687E 7028373N | 283 | 63.3°N | 1996 | (-/-) |
| Trøndelag | Hoklingen | Levanger | 308293E 7060454N | 88 | 63.6°N | 2014 | (M/-) |
| Trøndelag | Hostovatnet* ^π^ | Orkland | 226754E 7017927N | 197 | 63.2°N | 1988 | (M/-) |
| Trøndelag | Høysjøen | Verdal | 348668E 7085072N | 221 | 63.9°N | 2018 | (M/B) |
| Trøndelag | Igltjønna | Rindal | 208183E 7005200N | 148 | 63.0°N | 2023 | (-/-) |
| Trøndelag | Jonsvatnet* ^π^ | Trondheim | 279152E 7033929N | 149 | 63.4°N | 1980 | (G/-) |
| Trøndelag | Kobberdammen | Trondheim | 264552E 7041518N | 288 | 63.4°N | 2017 | (M/-) |
| Trøndelag | Kyvatnet | Trondheim | 267391E 7039499N | 183 | 63.4°N | 2016 | (-/-) |
| Trøndelag | Langhalstjønna | Meråker | 351921E 7025828N | 444 | 63.2°N | 2023 | (-/-) |
| Trøndelag | Langåsdammen* | Levanger | 318785E 7069844N | 134 | 63.7°N | 2014 | (G/-) |
| Trøndelag | Leirsjøen/Stor-Leirsjøen | Trondheim | 264311E 7037085N | 198 | 63.2°N | 2020 | (G/-) |
| Trøndelag | Leksdalsvatnet | Steinkjer/Verdal | 333242E 7084452N | 68 | 63.8°N | 2011 | (G/-) |
| Trøndelag | Lianvatnet | Trondheim | 266198E 7038786N | 221 | 63.4°N | 2012 | (-/-) |
| Trøndelag | Lømsen | Steinkjer | 330192E 7112680N | 39 | 64.1°N | 2014 | (G/-) |
| Trøndelag | Movatnet* | Levanger | 311155E 7060869N | 88 | 63.6°N | 2008 | (G/B) |
| Trøndelag | Nydammen/Langdammen/Noa | Trondheim | 265224E 7043071N | 218 | 63.3°N | 2023 | (G/-) |
| Trøndelag | Reinsvatnet | Steinkjer | 333171E 7105795N | 19 | 64.0°N | 2014 | (G/-) |
| Trøndelag | Ringavatnet | Orkland | 225543E 7016227N | 197 | 63.1°N | 2021 | (M/-) |
| Trøndelag | River Driva | Oppdal | 224378E 6947921N | 501 | 62.3°N | 2021 | (-/-) |
| Trøndelag | River Sandøla | Grong | 371210E 7150390N | 56 | 64.3°N | 2011 | (-/-) |
| Trøndelag | River Stjørdalselva | Stjørdal | 303183E 7043058N | 10 | 63.5°N | 2018 | (-/-) |
| Trøndelag | Romsjøen | Stjørdal | 304947E 7036448N | 186 | 63.2°N | 2022 | (VG/-) |
| Trøndelag | Rovatnet | Heim | 201060E 7027743N | 13 | 63.2°N | 2021 | (G/B) |
| Trøndelag | Rørvatnet | Rindal | 218111E 7011343N | 308 | 63.1°N | 2011 | (G/-) |
| Trøndelag | Selbusjøen | Selbu/Trondheim | 296502E 7017775N | 158 | 63.2°N | 2018 | (M/B) |
| Trøndelag | Skardsjøen | Stjørdal | 301700E 7047978N | 156 | 63.5°N | 2014 | (VG/-) |
| Trøndelag | Skjærsjøen | Steinkjer | 326730E 7122693N | 213 | 64.1°N | 2023 | (G/-) |
| Trøndelag | Snåsavatnet* | Snåsa | 355496E 7121275N | 23 | 64.2°N | 1982 | (M/B) |
| Trøndelag | Stordalsvannet | Åfjord | 270026E 7102107N | 18 | 64.0°N | 2018 | (VG/-) |
| Trøndelag | Stor-Drakstsjøen | Malvik | 286926E 7027401N | 263 | 63.3°N | 2013 | (G/-) |
| Trøndelag | Storsandtjønna | Røros | 314316E 6957310N | 659 | 62.7°N | 2014 | (-/-) |
| Trøndelag | Store Tyldvatnet | Stjørdal | 314849E 7051890N | 218 | 63.3°N | 2023 | (-/-) |
| Trøndelag | Storvatnet | Indre Fosen | 262011E 7063636N | 132 | 63.7°N | 2007 | (G/-) |
| Trøndelag | Svorksjøen* | Melhus/Orkland | 242237E 7010991N | 235 | 63.1°N | 2014 | (VG/-) |
| Trøndelag | Sørungen | Selbu | 295551E 7005818N | 454 | 63.1°N | 2011 | (M/-) |
| Trøndelag | Søvatnet | Heim/Orkland/Rindal | 217611E 7022621N | 280 | 63.2°N | 2014 | (G/-) |
| Trøndelag | Teksdalsvatnet | Ørland | 249959E 7088188N | 49 | 63.8°N | 2012 | (G/G) |
| Trøndelag | Theisendammen | Trondheim | 267783E 7041026N | 153 | 63.4°N | 2013 | (M/-) |
| Trøndelag | Tronestjønna | Namsskogan | 397850E 7181894N | 140 | 64.7°N | 2014 | (-/-) |
| Trøndelag | Tømmerholtdammen* | Trondheim | 274209E 7037117N | 246 | 63.4°N | 2008 | (G/-) |
| Trøndelag | Utnesvatn | Orkland | 239035E 7051531N | 77 | 63.5°N | 1981 | (G/-) |
| Trøndelag | Våddån | Skaun | 251422E 7020552N | 183 | 63.1°N | 2020 | (G/-) |
| Trøndelag | Øyvatnet | Høylandet | 372980E 7180890N | 62 | 64.7°N | 2014 | (M/G) |
| Trøndelag | Ånøya | Melhus/Skaun | 255946E 7017757N | 149 | 63.2°N | 2017 | (M/-) |
| Vestfold | Andredammen/2.dammen | Tønsberg | 238779E6580517N | 49 | 59.3°N | 2014 | (-/-) |
| Vestfold | Eikeren* ^π^ | Holmestrand (Øvre Eiker i Buskerud) | 214166E 6624625N | 19 | 59.6°N | 1982 | (M/B) |
| Vestfold | Farris/Farrisvannet | Larvik/Porsgrunn (Siljan i Telemark) | 213935E 6557687N | 23 | 59.2°N | 2012 | (G/G) |
| Vestfold | Goksjø | Sandefjord/Larvik | 222408E 6569642N | 28 | 59.2°N | 2014 | (VB/-) |
| Vestfold | Hajeren | Holmestrand (Kongsberg i Buskerud) | 213825E 6613759N | 411 | 59.6°N | 2012 | (M/-) |
| Vestfold | Holmsvannet/Revovannet | Holmestrand/Tønsberg | 226262E 6601284N | 46 | 59.3°N | 2022 | (VB/-) |
| Vestfold | Husvannet | Larvik | 213617E 6575574N | 33 | 59.2°N | 2018 | (-/-) |
| Vestfold | Langvannet | Tønsberg | 227334E 6589086N | 145 | 59.4°N | 2019 | (-/-) |
| Vestfold | Skjærsjøen | Larvik | 210537E 6572788N | 80 | 59.2°N | 2014 | (-/-) |
| Vestfold | Ulfsbakktjern | Larvik | 211744E 6556096N | 135 | 59.0°N | 2020 | (VG) |
| Vestfold | Vikevannet* | Holmestrand | 223708E 6610591N | 36 | 59.5°N | 1997 | (B/-) |
| Vestland | Byrkjelandsvatnet | Alver | -43798E 6765708N | 30 | 60.4°N | 2020 | (-/-) |
| Vestland | Bjørnevatnet | Bergen | -31729E 6722446N | 214 | 60.2°N | 2020 | (-/-) |
| Vestland | Borgavatnet | Osterøy | -20969E 6746256N | 33 | 60.2°N | 2019 | (B/-) |
| Vestland | Gravdalsvatnet | Bergen | -35534E 6733724N | 13 | 60.2°N | 2022 | (M/-) |
| Vestland | Hafslovatnet* | Luster | 81198E 6821428N | 168 | 61.3°N | 1991 | (G/-) |
| Vestland | Haukelandsvatnet | Bergen | -24817E 6730088N | 72 | 60.2°N | 2020 | (M/B) |
| Vestland | Hopsvatnet | Masfjorden | -11835E 6790994N | 2 | 60.6°N | 2020 | (G/-) |
| Vestland | Håheimsvatnet* | Sunnfjord | 50126E 6858015N | 198 | 61.6°N | 2010 | (-/-) |
| Vestland | Kalandsvatnet* | Bergen | -30044E 6720647N | 53 | 60.3°N | 2010 | (M/-) |
| Vestland | Kiplevatnet | Bergen | -38905E 6732693N | 42 | 60.4°N | 2019 | (-/-) |
| Vestland | Klokkarvatnet | Bergen | -31815E 6720097N | 53 | 60.2°N | 2020 | (-/-) |
| Vestland | Knappatjørna | Bergen | -36372E 6727742N | 45 | 60.2°N | 2020 | (G/G) |
| Vestland | Rebnorsvatnet* | Austrheim | -49907E 6781619N | 9 | 60.5°N | 1995 | (-/-) |
| Vestland | Røldalsvatnet | Ullensvang | 39703E 6658577N | 380 | 59.8°N | 2018 | (G/-) |
| Vestland | Signalsvatnet | Øygarden | -49869E 6730084N | 105 | 60.2°N | 2023 | (G/-) |
| Vestland | Småvatnet | Bergen | -39350E 6730992N | 20 | 60.2°N | 2020 | (VG/-) |
| Vestland | Storavatnet | Bergen | -38499E 6731120N | 40 | 60.3°N | 2019 | (G/G) |
| Vestland | Storavatnet/Vestrevatnet* | Osterøy/Vaksdal | -15742E 6750074N | 153 | 60.6°N | 1982 | (G/-) |
| Vestland | Storavatnet | Stord | -36447E 6665817N | 11 | 59.8°N | 2012 | (M/-) |
| Vestland | Strynevatnet/Oppstrynsvatnet | Stryn | 77773E 6893173N | 29 | 61.9°N | 2011 | (G/G) |
| Vestland | Tennebekktjern | Bergen | -35877E 6731799N | 95 | 60.4°N | 2014 | (G/-) |
| Vestland | Tranevatnet | Bergen | -31527E 6724279N | 53 | 60.2°N | 2023 | (G/-) |
| Vestland | Tveitavatnet | Alver | -28346E 6762623N | 23 | 60.6°N | 2014 | (M/-) |
| Vestland | Vetlavatnet | Kvam | 20314E 6731169N | 749 | 60.3°N | 2012 | (-/-) |
| Vestland | Vigdarvatnet | Sveio (Haugesund i Rogaland) | -44039E 6634828N | 8 | 59.3°N | 2019 | (M/B) |
| Vestland | Ulvesetvatnet | Øygarden | -48601E 6728488N | 16 | 60.3°N | 2012 | (-/-) |
| Vestland | Ådlandsvatnet | Stord | -32818E 6666901N | 4 | 59.8°N | 1997 | (M/-) |
| Østfold | Aremarksjøen | Aremark | 310155E 6573603N | 105 | 59.1°N | 2023 | (M/-) |
| Østfold | Bergsjøtjernet | Skiptvet | 276618E 6602216N | 152 | 59.3°N | 2022 | (-/-) |
| Østfold | Bjørndalsdammene | Fredrikstad | 268902E 6573052N | 57 | 59.1°N | 2021 | (-/-) |
| Østfold | Børtevannet | Sarpsborg | 289463E 6576149N | 86 | 59.2°N | 2020 | (-/-) |
| Østfold | Femsjøen | Halden | 299734E 6562310N | 80 | 59.1°N | 2021 | (M/-) |
| Østfold | Holtetjern | Rakkestad | 293853E 6576355N | 136 | 59.2°N | 2023 | (-/-) |
| Østfold | Lundebyvannet | Indre Østfold | 301161E 6606460N | 158 | 59.3°N | 2022 | (B/G) |
| Østfold | Nordre Kornsjø | Halden | 308801E 6539547N | 141 | 58.9°N | 2015 | (G/-) |
| Østfold | Lyseren | Indre Østfold (Enebakk i Akershus) | 280413E 6623327N | 161 | 59.7°N | 2018 | (G/B) |
| Østfold | Rødenessjøen | Marker | 308075E 6607723N | 119 | 59.3°N | 2020 | (M/-) |
| Østfold | Tunevannet | Sarpsborg | 277452E 6580785N | 40 | 59.3°N | 2013 | (B/B) |
| Østfold | Vansjø | Råde/Moss/Våler | 264692E 6590498N | 25 | 59.4°N | 2017 | (M/-) |
| Østfold | Vientjern | Indre Østfold (Nordre Follo i Akershus) | 272499E 6623490N | 183 | 59.7°N | 2018 | (-/-) |
| Østfold | Øymarksjøen | Marker | 310182E 6588159N | 115 | 59.2°N | 2023 | (M/-) |

* Lakes where cercarial dermatitis is reported frequently (almost every year).

π Lakes where ocellate cercaria have been found.

! *Trichobilharzia franki* identified from a *Radix auricularia* (cf. Soleng A, Mehl R 2011 https://doi.org/10.1017/S0022149X10000672)
